# Analysis of a Multi-Environment Trial for Black Raspberry (*Rubus occidentalis* L.) Quality Traits

**DOI:** 10.3390/genes13030418

**Published:** 2022-02-25

**Authors:** Matthew R. Willman, Jill M. Bushakra, Nahla Bassil, Chad E. Finn, Michael Dossett, Penelope Perkins-Veazie, Christine M. Bradish, Gina E. Fernandez, Courtney A. Weber, Joseph C. Scheerens, Lisa Dunlap, Jonathan Fresnedo-Ramírez

**Affiliations:** 1 Department of Horticulture and Crop Science, The Ohio State University, Wooster, OH 44691, USA; willman.19@osu.edu (M.R.W.); scheerens.1@osu.edu (J.C.S.); dunlap.352@osu.edu (L.D.); 2 National Clonal Germplasm Repository, United States Department of Agriculture-Agricultural Research Service (USDA-ARS), Corvallis, OR 97333, USA; jill.bushakra@usda.gov (J.M.B.); nahla.bassil@usda.gov (N.B.); 3 Horticultural Crops Research Unit, United States Department of Agriculture-Agricultural Research Service (USDA-ARS), Corvallis, OR 97333, USA; chad.finn@usda.gov; 4 BC Berry Cultivar Development Inc., Abbotsford, BC V2T 1W5, Canada; r.idaeus@gmail.com; 5 Department of Horticultural Science, North Carolina State University, Raleigh, NC 27695, USA; penelope_perkins@ncsu.edu (P.P.-V.); cmbradis@ncsu.edu (C.M.B.); gefernan@ncsu.edu (G.E.F.); 6 Cornell AgriTech, New York State Agricultural Experiment Station, Geneva, NY 14456, USA; caw34@cornell.edu

**Keywords:** high-throughput genotyping, pomological traits, mixed model analysis, genotype-by-environment interaction, QTL-by-environment interaction

## Abstract

U.S. black raspberry (BR) production is currently limited by narrowly adapted, elite germplasm. An improved understanding of genetic control and the stability of pomological traits will inform the development of improved BR germplasm and cultivars. To this end, the analysis of a multiple-environment trial of a BR mapping population derived from a cross that combines wild ancestors introgressed with commercial cultivars on both sides of its pedigree has provided insights into genetic variation, genotype-by-environment interactions, quantitative trait loci (QTL), and QTL-by-environment interactions (QEI) of fruit quality traits among diverse field environments. The genetic components and stability of four fruit size traits and six fruit biochemistry traits were characterized in this mapping population following their evaluation over three years at four distinct locations representative of current U.S. BR production. This revealed relatively stable genetic control of the four fruit size traits across the tested production environments and less stable genetic control of the fruit biochemistry traits. Of the fifteen total QTL, eleven exhibited significant QEI. Closely overlapping QTL revealed the linkage of several fruit size traits: fruit mass, drupelet count, and seed fraction. These and related findings are expected to guide further genetic characterization of BR fruit quality, management of breeding germplasm, and development of improved BR cultivars for U.S. production.

## 1. Introduction

Black raspberry (*Rubus occidentalis* L.) is a high-value yet minor crop cultivated in the U.S. for both fresh and processing markets. Black raspberry (BR) is endemic to eastern North America, where it is enjoyed both as wild forage and in commercially cultivated varieties. To date, it is commercially grown as a specialty crop in the Midwest, Northeast, and Northwest regions of the U.S. There is also active interest in expanding BR production into the southern U.S. [1]. 

BR is also used as a source of genetic variation for red raspberry (*Rubus idaeus* L.) breeding to improve adaptation and other commercial traits, although the trend among consumers is a preference for European red raspberry fruit [2]. BR fruit tend to be small and seedy, whereas fresh fruit consumers prefer large fruit with smaller seeds (C.E. Finn, personal observation). In red raspberry, selection for increased drupelet count and drupelet size have resulted in larger fruit and increased yields [3]. However, breeding attempts to increase BR fruit size have historically been met with limited success [4,5,6], limiting the availability of diverse productive cultivars to growers [7,8]. 

Resurging interest in BR as a high-value crop has led to increased interest in BR genetics, following discoveries of potential health benefits associated with its fruit [9,10] and a general increase in U.S. demand for fresh small fruit [11]. A study of diverse BR breeding germplasm found high genetic variance for fruit chemistry properties (i.e., pH, titratable acidity, percent soluble solids, anthocyanin profiles, and total anthocyanin), cane vigor, flowering time, and ripening time [12]. A recent study of BR anthocyanin content has followed interest in dietary anthocyanins for their health-promoting characteristics [13], as anthocyanins are responsible for the dark coloration of BR fruit. Genetic variation for anthocyanin content in wild BR accessions has been reported [14,15,16]. Total anthocyanin content and total phenolic content were found to vary significantly between progeny groups of controlled BR crosses [14]. BR accessions lacking anthocyanidin-3-rutinoside, a major component of the BR anthocyanin modification pathway, have also been described [16]. These mutants were found to have elevated cyanidin-3-sambubioside proportions and low total anthocyanin content [16].

Production environment has also been shown to influence BR biochemistry. A comparison of BR cultivars grown in multiple Ohio production sites found significant differences in antioxidant capacity, anthocyanin content, and titratable acidity (TAc) between production sites [17]. In addition, this study found no significant differences in antioxidant capacity, anthocyanin content, or titratable acidity between the three tested cultivars [17]. A multi-environment trial (MET) using BR germplasm grown at research stations in Oregon, Ohio, North Carolina, and New York found statistically significant differences in soluble solid content (SSC), pH, TAc, and SSC:TAc among research locations as well as among genotypes [18]. Differential performances of fruit firmness and vigor were reported for some experimental BR genotypes trialed in Oregon, Ohio, New York, and North Carolina, though no genotype-by-environment interaction (GEI) statistics were reported [19]. A summary of GEI is available for the primocane-fruiting trait, though the GEI of this trait remain poorly understood [20].

Challenges in BR breeding brought on by a lack of elite genetic diversity could have dissuaded public and private breeding operations from investing resources in BR improvement. According to a 2002 report on *Rubus* breeding, of 30 *Rubus* breeding programs spanning 19 countries, only 7 of these were actively breeding BR, and none were pursuing BR as their main crop [21]. Only five BR cultivars have been released in the U.S. since 1980: ‘Earlysweet’, ‘Haut’, ‘Ohio’s Treasure’ (U.S. Plant Pat. No. 27,871), ‘Explorer’ (U.S. Plant Pat. No. 17,727), and ‘Niwot’ (U.S. Plant Pat. No. 27,131). In the same timeframe, over 100 red raspberry cultivars were released [21].

Commercial production is currently constrained by a lack of high-performing cultivars, stemming from low genetic diversity within elite germplasm. In fresh markets, production is currently limited by a lack of cultivars meeting consumer preference demands, including large fruits with a small seed fraction. In both fresh and processing markets, production is limited by insufficient yields and poor planting longevity. The improvement of BR germplasm will require the identification and consolidation of genetic components influencing multiple fruit quality traits. Current strategies for BR breeding include crossing of selections from the wild and using germplasm constructed from wild and cultivated genotypes [22,23]. 

GEI is a particular concern in perennial fruit crops, where breeding efforts are often restricted by long juvenile periods, self-incompatibility, susceptibility to inbreeding depression, and selection of many complex traits [24]. Cultivar development of a perennial fruit crop is expected to require ten years at minimum following the final cross [24]. Thus, it is important that final selections have high genetic merit and stable trait expression across production environments. Multiple methods exist for the analysis of GEI [25], and they allow for the identification of stable and unstable genetic components that may be worth selection. Due to high costs associated with establishment and maintenance, METs of perennial crops are often unbalanced and lack within-environment replication. Mixed effects models optimize the use of unbalanced data and allow for the estimation of complex variance-covariance structures for random effects [26]. 

The estimation of GEI through mixed model analysis of METs is becoming increasingly common in perennial crops [27,28,29,30]. These studies enable the identification of high-performing and stable individuals as well as breeding value predictions for unbalanced METs. Since BR is cultivated in diverse U.S. geographic regions and given the limited resources for BR breeding, the efficient assessment of breeding germplasm among diverse production environments is paramount to successful genetic improvement of this crop. GEIs are observed as differential genetic responses of accessions grown in heterogeneous environments via a MET, which today can be enhanced by the implementation of high-throughput genotyping and the application of mixed-model quantitative trait locus (QTL) mapping [31,32]. 

The main objective of this study is to elucidate genetic contributors to fruit quality in BR. Such elucidation will improve our understanding of BR biology and genetics and enable the development of strategies for breeding. The specific objectives are to (i) estimate and describe genotype-by-environment interactions within diverse U.S. locations for two mapping populations, each having wild and cultivated ancestry, and (ii) discover QTL contributing to horticulturally relevant traits within these populations across and within the environments described below. Finally, concluding remarks are provided with respect to possible future avenues of research regarding BR.

## 2. Materials and Methods

### 2.1. Germplasm

Two F_1_ mapping populations, ORUS 4304 and ORUS 4305, were constructed through controlled pollination in Corvallis, Oregon, and were composed of 192 and 115 individuals, respectively. Founders for both populations included the fresh market cultivar, ‘Jewel’ [33] and the wild accession, NC 84-10-3 [12]. Wild selections ORUS 3817-2 and ORUS 3778-1 are founders for ORUS 4304 and ORUS 4305, respectively [20].

### 2.2. Phenotypic Data

ORUS 4304 and ORUS 4305 progeny, parents, and common ancestor, ‘Jewel’, were planted at four U.S. locations (Corvallis, Oregon (OR); Wooster, Ohio (OH); Jackson Springs, North Carolina (NC); and Geneva, New York (NY)) in the spring of 2012. Fertilizer, pesticides, and irrigation were applied according to local recommendations. In all locations, first-year canes were cut to ~1 m in length each summer to promote lateral branch growth.

Ripe fruit were hand-collected at trial locations in the summers of 2013, 2014, and 2015 in a total of eleven location-by-year environments. Fruit were determined to be ripe once they were fully colored and could be easily removed from the torus. Collected fruit were frozen and sent to Kannapolis, North Carolina, where they were analyzed for chemical and physical fruit quality traits: titratable acidity (TAc, % citric acid equivalents), pH, SSC (°Brix), total anthocyanin content (AnC, mg/kg cyanidin 3-glucoside equivalents), total phenolics content (PhC, mg/kg gallic acid equivalents), average infructescence (fruit) mass (FrM), and average seed mass (SdM) [18]. SSC and TAc were used to calculate the ratio, SSC:TAc. Drupelet count (DrC) was calculated as the number of seeds per fruit, assuming one seed per druplet. Drupelet mass (DMs) was calculated as fruit mass per drupelet count. Seed fraction (SFr) was calculated as seed mass per fruit mass.

Most individuals (parents and progeny) were replicated across locations; however, not all individuals were replicated across trial locations, resulting in unbalanced phenotypic data among locations. Further, because of differences in plant health, phenotypic data collected within locations were incomplete across trial years. Progeny individuals were not replicated within locations. Fruit and seed size traits were not recorded in Oregon in 2013. Poor plant performance in 2015 resulted in no fruit collection in North Carolina.

### 2.3. Genotyping and Relatedness Analysis

DNA was extracted, libraries were prepared, and genotyping-by-sequencing (GBS) data were collected in three separate batches for a total of seven sequencing runs. Due to logistic situations, four GBS runs were completed in Oregon, two in North Carolina, and one in Ohio. Initial GBS data were collected in Oregon and North Carolina as described by Bushakra et al. in 2018 [23]. Preliminary analysis of these data revealed low sequence depth for 145 individuals; therefore, additional sequencing data were collected in Ohio via a single GBS run with 72 available individuals of the 145 that had low depth. In Ohio, leaf samples were collected, lyophilized, and held in dry storage before DNA extraction. Leaf tissue was ground by mortar and pestle, and DNA was extracted using a modified CTAB protocol [34]. Following RNase treatment, DNA was further purified using the Genomic DNA Clean & Concentrator-10 kit (Zymo Research, Irvine, CA, USA). Genomic DNA concentration and quality were initially assessed using a NanoDrop ND-1000 spectrophotometer (NanoDrop Technologies, Wilmington, DE, USA) and subsequently assessed using a Qubit 4 fluorometer (Thermo Fisher, Waltham, MA, USA) and via gel electrophoresis.

In Ohio, genomic DNA samples were submitted to the Ohio State University Molecular and Cellular Imaging Center (Wooster, OH, USA) for GBS library construction and sequencing. GBS libraries were constructed following Elshire et al. [35]. Briefly, genomic DNA samples were digested with *Ape*KI and ligated to adapters compatible with *Ape*KI sticky ends and containing unique barcodes with partial Illumina^®^ adaptor sequences. Following PCR amplification using Illumina^®^ Nextera adapters (Illumina, San Diego, CA, USA), libraries were pooled and quantified using a Qubit^®^ fluorometer (Thermo Fisher, Waltham, MA, USA), and size distribution and concentration were confirmed using the 4200 TapeStation (Agilent Technologies, Santa Clara, CA, USA). The pooled library was analyzed using single-read sequencing of 151 cycles on the NextSeq (Illumina, San Diego, CA, USA) instrument at the HudsonAlpha Institute for Biotechnology (Huntsville, AL, USA).

All GBS data, representing 305 individuals, were analyzed simultaneously. Data were initially subjected to quality control and adapter removal using FastQC v. 0.11.7 [36] and Trimmomatic v. 0.36 [37]. Single-nucleotide polymorphisms (SNPs) were identified using the TASSEL 5 GBS v2 pipeline v. 5.2.48 [38] and the BR reference genome sequence V.3 [39]. The SNP filtering criteria consisted of a minor allele frequency of 0.01, minimum sequence depth per individual of 6, maximum missing alleles frequency of 0.80, and a thinning interval of 100,000 bp. SNP-guided relationship analyses were conducted separately for populations ORUS 4304 and ORUS 4305 using VCFtools v. 1.16 [40] and the method described by Yang et al. [41]. Estimated relationship statistics were then checked against pedigree population structure to confirm the relatedness of population progeny.

### 2.4. Phenotypic Analysis and Determination of Variance–Covariance Structure

Phenotypic data were checked for obvious outliers for each trait-by-environment combination via the visual examination of scatter plots using R software v 3.5.1 [42]. Phenotypic data were checked for homoscedasticity for each trait among environments via the evaluation of residuals in a fixed effect analysis of variance (ANOVA).

To estimate within-environment variance parameters, multiple genomic and residual variance structures were fit for each phenotypic trait using ASReml v. 4.1 [43] and statistical methods described by Hardner et al. [28]. Briefly, a general mixed model was fit with fixed location-by-year environment effects and random genomic-by-environment effects. The variation of observations were assumed to follow a multivariate normal distribution. To account for unbalanced phenotypic data (i.e., missing individual-by-environment observations), an additive relationship matrix (G_A_) matrix was generated using the R package ‘synbreed’ v 0.12.9 [44] and the method described by [45] and incorporated into the model.

Additive genomic-by-environment variance–covariance matrix (G_A×E_) structures and residual variance–covariance matrix (R) structures were modeled as described by [46]. Four models were fit for each trait (Table 1). Briefly, G_A×E_ structures were fit as homogeneous with uniform correlation (Model 1), heterogeneous with uniform correlation (Models 2 and 3), and heterogeneous with non-uniform correlation estimated via factor analysis (Model 4). Residual variance structures were fit as independent with homogeneous variance (Models 1 and 2) and independent with heterogeneous variance (Models 3 and 4). The most parsimonious model (i.e., a model with as few explanatory terms possible while conserving the greatest explanatory power) was selected for all traits via likelihood ratio tests [46].

Narrow-sense heritability was estimated within each environment as Equation (1):

(1) $$h_{j}^{2}=\frac{\sigma_{A_{j}}^{2}}{\sigma_{A_{j}}^{2}+\sigma_{R_{j}}^{2}}$$

where $\sigma_{A_{j}}^{2}$ and $\sigma_{R_{j}}^{2}$ were the estimated additive genomic and residual variances, respectively, for the *j* th location-by-year environment.

To explore correlation between traits, individual breeding values (BVs) were predicted across environments via the best linear unbiased prediction of individual genetic effects. Pairwise Pearson correlation coefficients were then estimated between BVs for all traits.

### 2.5. Linkage Mapping and QTL Analysis

For linkage map construction and QTL analysis, SNPs were filtered for high confidence and low incidence of missing values among individuals using VCFtools v. 1.16 [40]. Passing SNPs were merged with simple sequence repeat (SSR) data collected by Bushakra [23] prior to linkage map construction.

#### 2.5.1. Linkage Map Construction

All markers were recoded to match outbreeding segregation types (lm × ll, nn × np, hk × hk, ef × eg, and ab × cd) as described by Van Ooijen and Jansen [47]. Expected segregation patterns are 1:1 for lm × ll and nn×np markers, 1:2:1 for hk × hk markers, and 1:1:1:1 for ef × eg and ab × cd markers. A Chi-square test was applied to each marker to test for segregation distortion, and markers with significant deviations from expected segregation patterns (*p* ≤ 0.05) were discarded.

Linkage maps were constructed using JoinMap v. 4.1 [48]. Missing genotypic data can result in erroneous marker placement; thus, individuals with ≥30% missing marker data were excluded from map construction and subsequent analysis. Due to a low number of conserved markers and a low density of markers per linkage group in ORUS 4304, maps were only constructed for ORUS 4305. Mapped individuals were selected based on the availability of phenotypic data for several traits, including but not limited to those described in the present study.

An Independence Likelihood of Odds (LOD) threshold of 10 was used to establish linkage groups (LG). For the determination of multipoint frequency estimation, ordering and map integration, loci within each LG were subjected to the maximum likelihood (ML) mapping procedure [49]. A nearest neighbor stress (N.N. stress) threshold of 5 cM was used to identify misplaced markers, which were removed. Remaining markers were again subjected to the ML algorithm, and this cycle was repeated until all markers fell below the N.N. stress threshold immediately following ML mapping. Seven maps were thus constructed representing the seven chromosomes of the BR genome.

#### 2.5.2. QTL Mapping

Multiple-environment linkage analysis was performed for each trait in ORUS 4305 using GenStat v. 19.1 routines: QMQTLSCAN, QMESTIMATE, and QMBACKSELECT and a default maximum step size of 1,000,000 cM [50]. Genetic (co)variance model selection was determined as the most parsimonious model identified via mixed model analysis.

Candidate QTL were first identified via simple interval mapping (SIM) by fitting the model using Equation (2):

(2) $$y_{ij}=\mu +E_{j}+\alpha_{j}^{a}x_{i}^{a}+\alpha_{j}^{a2}x_{i}^{a2}+\alpha_{j}^{d}x_{i}^{d}+\varepsilon_{ij}+e_{ij}$$

where $y_{ij}$ is the trait mean for genotype *i* in environment *j*, μ is the overall mean, $E_{j}$ is the environment *j* main effect, $\alpha_{j}^{a}$ is the additive QTL effect for environment *j* at the position being tested, $x_{i}^{a}$ is the additive genetic predictor for genotype *i* at the position being tested, $\alpha_{j}^{a2}$ is the additive QTL effect of the second parent for environment *j* at the position being tested, $x_{i}^{a2}$ is the additive genetic predictor of the second parent for genotype *i* at the position being tested, $\alpha_{j}^{d}$ is the dominance QTL effect for environment *j* at the position being tested, $x_{i}^{d}$ is the dominance genetic predictor for genotype *i* at the position being tested, $\varepsilon_{ij}$ is the genetic residual for genotype *i* in environment *j*, and $e_{ij}$ is the unit error for genotype *i* in environment *j*.

Significance thresholds were determined using the Genstat^®^ default method of Li and Ji [51]. Candidate QTL identified via SIM were fit as cofactors by composite interval mapping (CIM). Cofactors falling below the significance threshold were removed, new candidate QTL were selected, and CIM was repeated until candidate QTL selection did not change. Candidate QTL identified via CIM were then fit to a final multiple-environment model by backward selection to estimate additive and dominance effects.

As indicated in Equation (2), additive effects were estimated for each parent separately. In a cross pollinated (CP) population, each locus comprises a maximum of four possible alleles, each contributed by one of four grandparents. Though the linkage phase of a CP population cannot be defined a priori (as it is in a population derived from inbred individuals), it must be estimated during linkage map construction. Thus, additive grandparental genetic contributions can be estimated within each CP parent. This is particularly relevant in cases where two or more QTL are identified on a single LG, as it may allow for the identification of linked genetic effects.

Following multiple-environment model selection in Genstat^®^, multiple-QTL, single-environment models were then fit for each trait using R/qtl v. 1.42.8 [52] to (i) estimate the proportion of variance explained by each QTL within each environment and (ii) estimate 95% Bayes Credible Intervals (BCIs) for each significant QTL-by-environment combination. Significance was determined for each QTL-by-environment combination via estimation of a point-wise *p*-value for each locus via drop-one ANOVA of a strictly additive QTL model. Loci with a *p*-value ≤ 0.05 were determined to be significant. Before BCI estimation, within-environment QTL positions were optimized using the refineqtl() function in R/qtl. The genomic position of each QTL region was then defined using the physical positions of markers flanking each BCI. QTL intervals were visualized using MapChart v2.32 [53].

QTL were named according to recommendations provided by the Genome Database for Rosaceae (GDR).

## 3. Results

### 3.1. Marker Data and Relatedness Analysis

**Figure 1:**
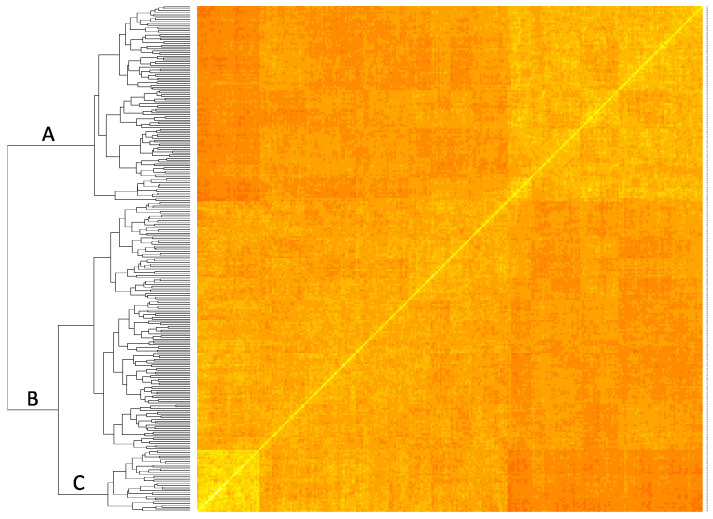
Additive relatedness matrix for ORUS 4304, ORUS 4305, and parents. Coefficients were estimated using the ‘AGHmatrix’ R package, VanRaden method, and 416 genome-wide markers. A = 4305; B = 4304; C = putative maternal inbred progeny.

A total of 242,391 SNPs were identified among 305 individuals from both populations using the TASSEL 5 GBS v2 pipeline. Following SNP-calling, 20 individuals were excluded from further analysis following an inability to call reliable marker genotypes due to low marker depth. Relatedness analysis of ORUS 4304 revealed two clusters and one additional outlier (Figure 1). One cluster containing 38 progeny showed close relatedness to the maternal parent, indicating these individuals likely resulted from self-pollination of the maternal parent. Relatedness analysis of ORUS 4305 indicated five off-types of unknown origin: 4305-38, 4305-39, 4305-41, 4305-59, and 4305-65.

A G_A_ matrix was estimated for 285 individuals (174 ORUS 4304 progeny, 107 ORUS 4305 progeny, and parents (3021-2, 4153-1, 4158-2), and a common grandparent (‘Jewel’)). To construct the G_A_ matrix, 427 high-confidence SNPs with low missing values among individuals were selected. The resulting matrix was used to define the variance–covariance of additive genomic effects in a mixed model analysis of all phenotypic traits.

### 3.2. Phenotypic Analysis and Determination of Variance–Covariance Structure

#### 3.2.1. Fruit Size Traits

Data sets for all traits were determined to be homoscedastic and normally distributed. For DrM, FrM, SdF, and SdM, a model fitting heterogeneous G_A×E_ variance with uniform correlation between environments and heterogeneous residual variance (Model 3) was found to be the most parsimonious model. For DrC, an extended factor analytic model which estimates non-uniform G_A×E_ correlation structures (Model 4) was found to be the most parsimonious model (*p* < 0.05, Table 2).

Genome-by-environment correlations were estimated for DrC, DrM, FrM, SdF, and SdM (Table 3). Correlations were highest for fruit mass (0.89, s.e. = 0.036) and lowest for seed fraction (0.79, s.e. = 0.065). 

Narrow-sense heritability estimates for each trait-by-environment combination are summarized in Table 4. Heritability ranged from 0.02 to 0.89. In general, the narrow-sense heritability of seed mass was high in all environments. However, seed fraction was highly variable, having lower values in Oregon and higher values in Ohio. 

#### 3.2.2. Fruit Chemistry Traits

For TAc and pH, a model fitting homogeneous G_A×E_ variance and heterogeneous residual variance (Model 2) was found to be the most parsimonious model. For AnC, SSC, and SSC:TAc, a model fitting heterogeneous G_A×E_ variance and heterogeneous residual variance (Model 3) was found to be most parsimonious. For PhC, an extended factor analytic model which estimates non-uniform G_A×E_ correlation structure (Model 4) was found to be the most parsimonious model (*p* < 0.05, Table 2). 

Genome-by-environment correlations were estimated for pH, TAc, SSC, and AnC (Table 3). Correlations were highest for TAc (0.79, s.e. = 0.046) and lowest for SSC (0.46, s.e. = 0.097). Heritability estimates for fruit chemistry traits for each trait-by-environment combination are summarized in Table 4. Heritability ranged from 0.01 to 0.71. Soluble solid content and phenolics content had highly variable heritability estimates, showing a trend in which phenotypic data from Oregon yielded lower heritability estimates for both traits. Titrable acidity showed moderate to high values across environments, with the lowest again in Oregon and the highest in Ohio.

**Figure 2:**
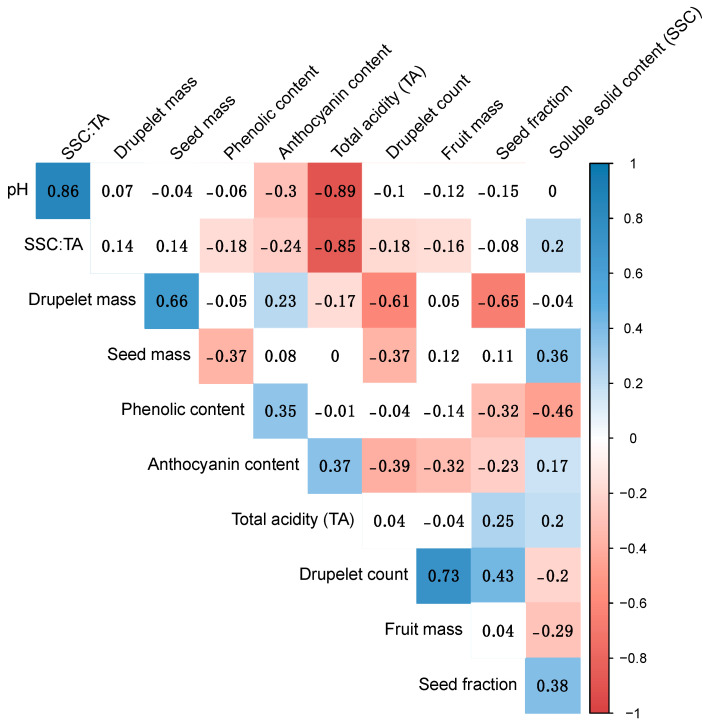
Pearson correlation coefficient plot of breeding values for fruit size and chemistry traits. Significant correlations (*p* < 0.01) are shaded in red (negative) or blue (positive).

Correlations between trait BVs are summarized in Figure 2 and Supplementary File 1. There were significant (*p* < 0.01) correlations between drupelet count and fruit mass (r = 0.74), drupelet count and seed fraction (r = 0.40), and drupelet count and seed mass (r = −0.32).

### 3.3. Genotyping and Linkage Map Construction

**Figure 3:**
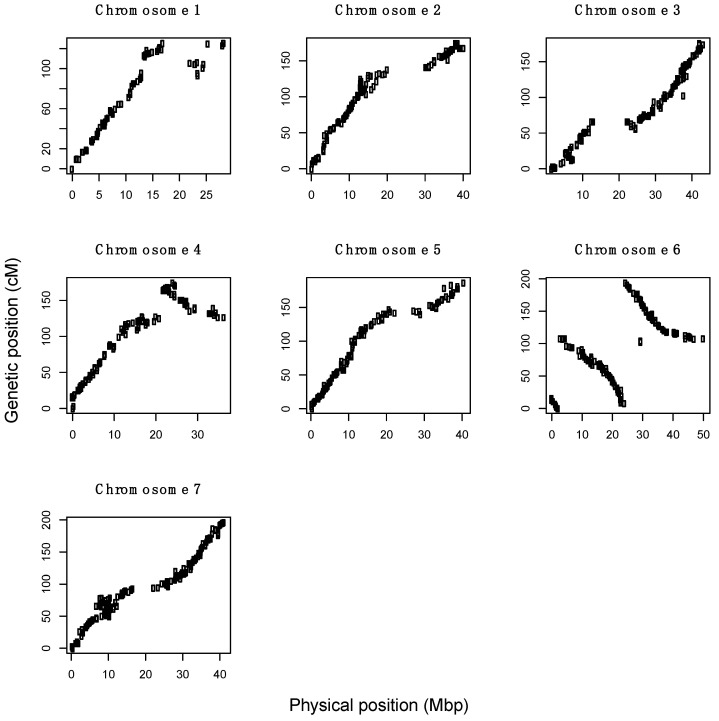
Genetic position plotted against physical genomic position for 974 combined SNP and SSR markers. A reduced slope and gap between groups of markers suggest reduced recombination and few markers near the centromere. Discontinuous portions in chromosomes 1, 4, and 6 suggest misalignments within either the linkage maps or the current genome assembly.

Prior to map construction, six individuals from ORUS 4305 were discarded due to a high number of markers with missing values (≥30%). Seven linkage maps were constructed spanning a total of 1230.7 cM and comprising 974 markers (931 SNP and 43 SSR) and 103 individuals. Linkage group lengths ranged from 128 to 196 cM and contained 96 to 171 markers. The collinearity of mapped genetic position to genomic position is illustrated in Figure 3. Discontinuous portions in chromosomes 1, 4, and 6 suggest misalignments within either the linkage maps or the current genome assembly. Based on recombination events in ORUS-4305, the current genome assembly of *R. occidentalis* needed additional scrutiny in these regions of chromosomes 1, 4, and 6.

### 3.4. QTL Mapping

Phenotypic data in ORUS 4305 were determined to be normally distributed for each trait-by-environment combination and homoscedastic for each trait among environments via the evaluation of residuals in a multivariate fixed effect ANOVA (Supplementary File 2, Figures S1–S10). Available phenotypic data for mapped individuals ranged from 47 to 71 observations per trait-by-environment combination (Supplementary File 2, Table S1). Of the 103 individuals included in the final linkage map, fruit size and fruit chemistry trait data were collected in one or more environments for 84 and 80 individuals, respectively.

Seventeen QTL were identified and associated with six of the nine traits analyzed (Table 5). Of these, QEI were significant for eleven QTL. Due to discontinuity between genetic and physical positions at LG 6 (Figure 3), genomic positions for putative QTL observed on LG 6 are not reported in this study.

#### 3.4.1. Fruit Size Traits

**Figure 4:**
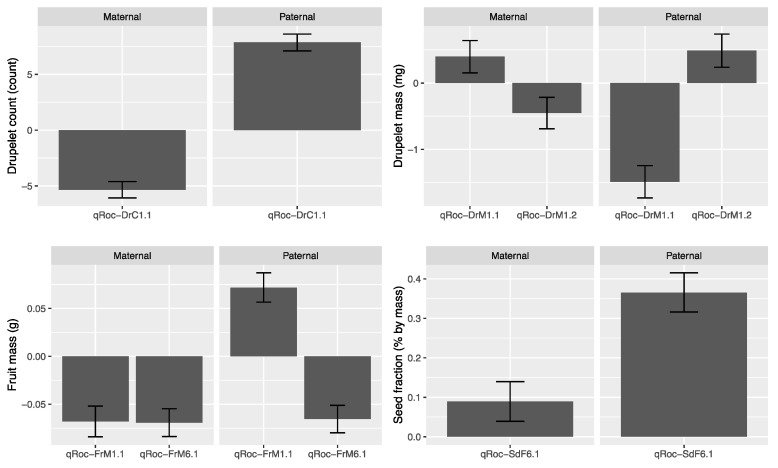
Additive effect size of stable QTL identified by multiple-environment analysis. Error bars indicate standard error.

Single-trait, multiple-environment linkage analysis was used to identify trait-associated regions for DrC, FrM, DrM, and SdF. No QTL were identified for SdM; however, SIM performed separately for each location-by-year environment found two SdM QTL on LGs 4 and 7 within environments OH 2015 and OH 2013, respectively. One or more fruit size QTL were identified on LGs 1, 2, 4, 6, and 7. Stable QTL over environments were identified for FrM (qRoc-FrM1.1 and qRoc-FrM1.6), DrC (qRoc-DrC1.1), DrM (qRoc-DrM1.1 and qRoc-DrM1.2), and SdF (qRoc-SdF6.1). The additive effect size of these traits according to parental origin are shown in Figure 4. These QTL are of special interest since their stability suggests that they could be targets for the development of markers for the implementation of marker-assisted selection. Interestingly, most of these QTL were located on chromosomes 1 and 6. While qRoc-DrM1.2 had a very low statistical significance (−log10(*p*) ~ 1.2), the signal for this QTL was stable across environments, and its contribution to the expression of the trait may be minor but consistent.

Unstable QTL or QTL with a QEI component over distinct environments were also identified for DrC (qRoc-DrC1.2 and qRoc-DrC4.1), DrM (qRoc-DrM6.1), FrM (qRoc-FrM1.2 and qRoc-FrM2.1), and SdF (qRoc-SdF2.1 and qRoc-SdF7.1). In general, these QTL showed statistical significance (−log10(*p*)) above 3.4. Interestingly, qRoc-DrC1.2 had a −log10(*p*) = 16.24, which is a higher significance than some stable QTL identified. In Figure 4, the additive effect size for these QTL is shown. The general trend is that paternal alleles contribute mostly negatively across environments, just with distinct orders of magnitude; a clear example is qRoc-DrM6.1. Maternal allele contribution varied, and in several environments, the contribution of these alleles might not be distinct from zero, similar to qRoc-DrM6.1, qRoc-FrM1.2 and qRoc-FrM2.1. 

#### 3.4.2. Fruit Chemistry

**Figure 5:**
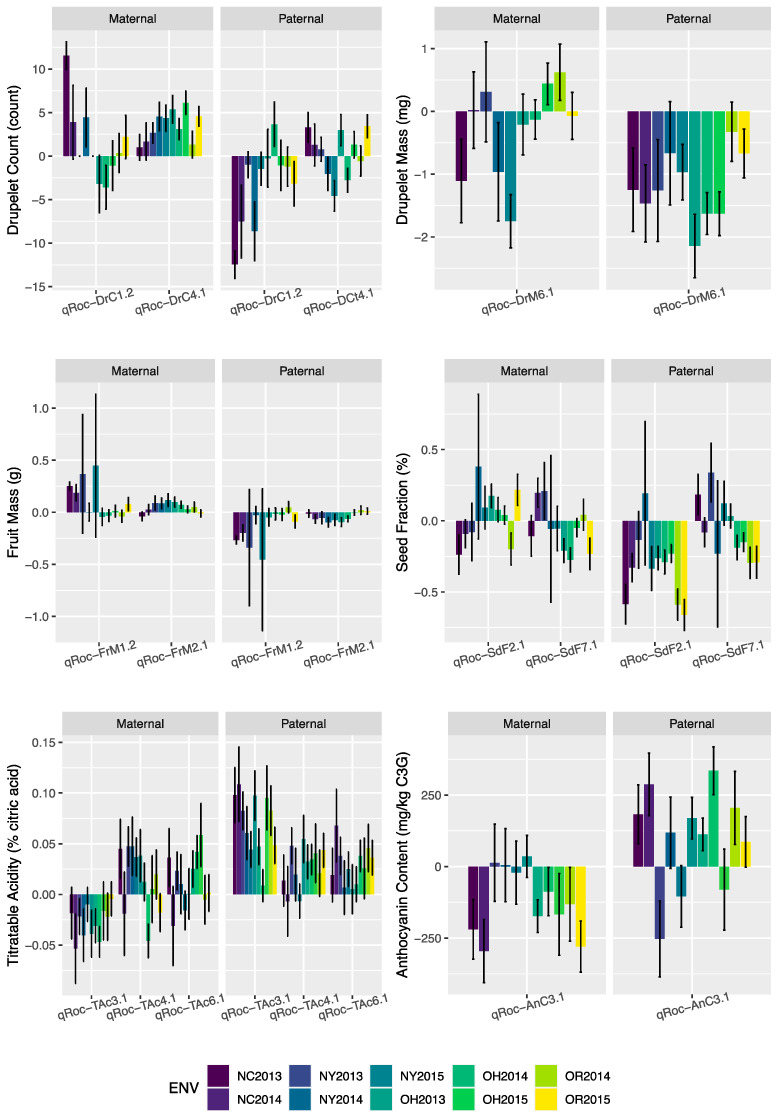
Additive effect size of unstable fruit morphology and fruit chemistry QTL identified by multiple-environment analysis.

Single-trait, multiple-environment linkage analysis was used to identify trait-associated regions for SSC, TAc, AnC, and PhC (Figure 5). No QTL were identified for SSC and PhC, which suggest higher complexity in comparison to other anthropogenic traits such as fruit-size-related traits. For Tac, three QTL were identified in LGs 3, 4, and 6 (qRoc-Tac3.1, qRoc-Tac4.1, qRoc-Tac6.1, respectively), and only one QTL on chromosome 3 for AnC (qRoc-AnC3.1). These QTL were identified with relatively low statistical significance (−log10(*p*) values below 6.5), and all of them had QEI, which is illustrated in Figure 4, in which the additive effect size for these QTL with respect to the parentals and each evaluated environment are shown. No obvious patterns are seen; however, for both TAc and AnC, it seems that the paternal alleles provide mostly positive effects across environments, with few exceptions. It is more evident for TAc, in which the effect of the maternal and paternal alleles was probably not different from zero in some environments. The counterpart is that the maternal alleles provide a range of negative effects in environments such as NC, OH, and OR, with NY probably not having an effect in the AnC phenotype. 

Given the complexity shown by TAc, additional single-environment analyses were performed in order to find which models, meaning how many and which QTL, better explain the TAc phenotype in each evaluated environment (Table 6). Thus, it was found that in OH in 2013 and 2015, up to three distinct QTL contributed to TAc. Interestingly, in 2013, two QTL on chromosome 4 contributed, according to the model, and both of them were far from the region in which qRoc-TAc4.1 was identified in the multi-environment trial. On the contrary, in NC, NY, and OR, usually, one QTL was the major contributor. For the single-environment combinations of NC 2012, NY 2014 and 2015, OH 2014, and OR 2014, the reported model was null, meaning that no significant QTL were found.

## 4. Discussion

In this study, fruit quality traits for eleven location-by-year trial environments were interrogated regarding (i) genome-by-environment correlations estimated across location-by-year environments, (ii) heterogeneous variance components and narrow-sense heritability coefficients estimated by environment, and (iii) QTL-by-environment interaction. GEIs for BR fruit traits are described here as genome-by-environment correlations and heritability coefficients arising from the estimation of heterogeneous genetic and residual variances. These estimates are informed by an SNP-derived G_A_ matrix, which allowed heterogeneous variance structures to be fit, although individuals were not replicated within trial locations [28]. 

### 4.1. Relatedness Analysis

The number of progenies identified as a result of self-pollination in ORUS 4304 in this study may explain the skewed segregation of the single-gene aphid resistance trait reported in ORUS 4304 in two studies [20,23]. Close relatedness to the maternal parent of the 38 ORUS 4304 off-types suggests that these individuals resulted from self-fertilization. Unlike some fruit crop species, black raspberry has been reported to produce self-compatible flowers [2], and no self-incompatibility mechanism in black raspberry has been described, further supporting the conclusion that progeny individuals with close resemblance to the maternal parent originated from self-pollination. The six ORUS 4305 identified in this study as off-types match those reported by Bushakra et al. [22] as having incongruous SNP data, and they were removed from linkage map construction in the cited study. 

The G_A_ matrix was constructed using a 427-count subset of 242,391 possible SNPs. This subset was defined by a series of filters selecting for markers that are biallelic, evenly distributed through the genome, and sequenced with high depth and low missing data among all individuals. This resulted in a less-than-typical number of markers used for genetic analysis. For example, 1617 markers selected from a 6k SNP array were used to estimate variance components of sweet cherry tested in eight environments [28]. However, individuals examined in the current study only include full siblings, half siblings, and three parents; thus, few recombination events (recent or ancestral) are expected among individuals, and kinship may be described by fewer, high-quality molecular markers than for studies with complex population structures.

### 4.2. Fruit Size Traits

Fruit size and its components, including drupelet count and seed size, are important traits for fresh market raspberry production. Current limitations to BR fresh market production include small fruit which are perceived by consumers as very seedy (C.E. Finn, personal observation). Increased fruit size and reduced seediness are important goals to the fresh BR industry. Breeding progress for increased fruit size and reduced seediness will rely on the identification and consolidation of genetic components contributing to these traits. 

Given interest in improving BR fresh fruit production within several U.S. geographical regions and limited resources for BR breeding, the efficient assessment of breeding germplasm among diverse production environments is paramount to successful genetic improvement of this crop.

Fruit mass and drupelet count are components of total yield and important qualities for fresh market production. High drupelet count has been associated with high yield in red raspberry cultivar development [3], and selection for high drupelet count may lead to larger BR fruit. Large fruit has also been associated with high yield in red raspberry and is an important trait for fresh market breeders and producers [3,54].

Several genes have been reported to control organ size in plant species, including *fw2.2* in tomato (*Solanum lycopersicum*) [55] and *Cell Number Regulator* (*CNR*) family genes in maize (*Zea mays*) [56]. CNRs are putative orthologs of *fw2.2*, and the expression of *CNR1* has been shown to reduce plant and organ size by reducing cell number [56]. This reduction in plant organ size is also strongly correlated with the copy number of *CNR1* [56]. CNR homologs have been identified in *Prunus* overlapping fruit size QTL in sweet cherry (*P. avium*) [57]. Preliminary BLAST alignment of *fw2.2* and *CNR1* proteins to the BR genome revealed homology between both proteins and the BR genome on chromosomes 2, 4, and 5, with hits on BR chromosome 4 falling within credible intervals for qRoc-DrC4.1, which may warrant further investigation.

Reduced seediness is an important goal in many fruit breeding programs, as evidenced by the fresh market popularity of seedlessness in grapes [58,59], citrus [60], and cucurbits [61]. Seediness is a particular concern in BR, as available cultivars are often perceived as very seedy, particularly in comparison to red raspberry. Selection for reduced seed mass and reduced seed fraction may generate BR accessions with reduced seediness. However, studies of perceived seediness in *Rubus* are sparse. The positive correlation between drupelet count and seed fraction suggests that increased drupelet count bears a cost of increased relative seed mass. This suggests a tradeoff to fresh market breeders and producers, as the fresh fruit market prefers large fruit with a small seed portion. The negative correlation between drupelet count and average seed mass suggests that increased drupelet production bears a cost of reduced seed size. This suggests a second tradeoff, more desirable than the first, as small seeds in high-drupelet fruit may be less recognizable than large seeds to the consumer.

Black raspberry has been noted as having a larger seed fraction than red raspberry for many years. Historically, the primary method of preservation of BR fruit was dehydration, which was generally done on the farm prior to sale [62]. While Card [62] noted that it was possible to dehydrate red raspberry for preservation, he indicates that it was rarely profitable to preserve the fruit this way, whereas the higher dry weights of BR made them more suited to this method. Moreover, Card also noted that the BR cultivars with the largest seeds were the most desired because these had the highest dry weight yield for the grower, since the production of dye was one of the main targets [62]. Though SdM is an important trait for *Rubus* breeding, SdF, calculated as a percentage of fresh seed mass over fresh fruit mass, may be a more important contributor to perceptible seediness. A 1931 study of *Rubus* fruit size and seediness traits found similar SdM between cultivated BR and red raspberry; however, SdF values observed for cultivated BR in this study were much greater (approximately twice) than those observed for cultivated red raspberry [63]. Such observations led Darrow and Sherwood [63] to suggest that proportion of seed weight to total berry weight is a more important seediness factor than seed size. The typical SdF observed in red raspberry breeding germplasm averages 4%, whereas the mean BR SdF observed in the present study was 8.0% (C.E. Finn, unpublished data).

SdF is related to FrM, as increased FrM may be achieved through increased seed or non-seed (i.e., flesh) portions of the fruit. Large fruits are desirable for fresh market production; however, increased FrM accompanied by increased seediness is undesirable. Two SdF QTL overlap QTL controlling FrM, one each on LGs 2 and 6, suggesting these traits may share pleiotropic or linked genetic components. High-value alleles for both traits originate from the same grandparent at the overlapping QTL on LG 2, indicating a positive correlation between FrM and SdF at this locus. Thus, selection for increased FrM at qRoc-FrM2.1 may bear a tradeoff of increased SdF. In contrast, high-value alleles originate from opposite grandparents at the overlapping QTL on LG 6, indicating a negative correlation between FrM and SdF at this locus. Thus, selection for increased FrM at qRoc-FrM6.1 may complement selection of reduced SdF. These findings emphasize that selection in BR for increased FrM should consider related seediness traits, including SdF.

Consumer panel testing in blackberry has shown a significant correlation between perceived seediness and seed fraction [64], and similar trends may be observed for BR. Contrasting this, there is limited evidence that seed mass may be a poor predictor of perceived seediness. For example, if seed mass is a good predictor of perceived seediness, one may expect cultivated red raspberry fruits to produce smaller seeds than those of BR. The average seed size in cultivated red raspberry grown in Corvallis, OR has been reported as 1.7 mg [65]. The average black raspberry seed mass of OR fruit in the present study is 1.8 mg, very similar to that reported for red raspberry. Though direct statistical comparison of these two studies cannot be conclusive, these observations suggest that perceived seediness may be influenced by factors aside from seed mass. 

Seediness in *Rubus* may be attributed to additional factors such as seed composition, and further study of seed physical traits (e.g., shape, density, and adherence to flesh) and chemical composition (e.g., lignin content) may elucidate these trends.

In BR, likely breeding targets include selection for high fruit mass, low seed mass, and low seed fraction. Thus, the selection of genotypes with high fruit mass and high drupelet count may coincide with the selection of unstable genotypes which perform especially well in high fruit mass and high drupelet count environments. On the other hand, selection for genotypes with low seed mass and low seed fraction may coincide with the selection of stable genotypes which perform relatively similarly in all environments.

### 4.3. Fruit Chemistry Traits

Fruit chemical composition in raspberry is greatly influenced by fruit maturity, with anthocyanin and sugar content increasing and acidity decreasing as fruit mature [66]. Fruit chemical composition traits in ripe raspberry fruits have been shown to be greatly influenced by environmental factors including location [17], harvest season [67], and photoperiod [68]. The current study presents three TAc QTL and one AnC QTL.

High GEI for phenolic content suggests that genetic and environmental components influencing this trait are complex. Raspberry fruit contains a large diversity of phenolic compounds including anthocyanins, phenolic acids, flavanols, and tannins [69]. In addition, the method used to measure phenolic content in the current study measures the abundance of all antioxidant compounds, including but not limited to phenolics [70].

Acidity is an important component of flavor in fruit crops [71]. Acidity levels are also important to processing fruit markets, as anthocyanin stability and color are both dependent on acidity and pH [72]. The production and accumulation of acidity in fruit is under the control of many complex factors [73]. The current study reports titratable acidity as % citric acid, as is common in raspberry studies [74,75]. A balance of sugar content and acidity is considered important for raspberry fruit flavor; however, genetic control of these traits tends to be unstable over different years and locations [69].

The effects of qRoc-TAc3.1 were significant in one or more years at all trial locations, suggesting its effect is fairly stable, and selection for increased or reduced acidity may be considered through phenotypic or marker assisted selection. This is relevant to the BR processing market, where increased fruit acidity may improve anthocyanin stability and the pigmentation of products containing BR extracts.

Candidate genes for titratable acidity have previously been mapped in peach [76]. In red raspberry, acid content has been found to vary between cultivars [67,77] and growing environments [67,68]. However, reported QTL associated with raspberry TAc were lacking.

Numerous anthocyanin compounds have been identified and characterized in *Rubus* fruit, including BR [78]. Dietary anthocyanins and other fruit phenolics are associated with improved human health, and important areas of research include determining mechanisms by which these phytochemicals influence human health and developing crop varieties with improved levels of beneficial compounds [79]. Anthocyanin biosynthesis enzymology and localization are well-characterized in model plant species; however, improved understanding of genetic regulation of anthocyanin biosynthesis in crop species is needed to develop crops with increased anthocyanin levels [80]. A wealth of genes regulating anthocyanin biosynthesis and the accumulation of anthocyanins have been discovered and characterized in Rosaceous small fruit, including cultivated strawberry (*Fragaria* × *ananassa*) [81,82,83,84] and wild strawberry (*F. vesca*) [85], red raspberry [86], and Korean black raspberry (*R. coreanus*) [87]. 

The AnC QTL identified on linkage group 3 in the present study may inform allelic variation for genetic components regulating anthocyanin biosynthesis and accumulation in BR. In 2009, Kassim et al. [88] reported major QTL for anthocyanin concentration in red raspberry in regions corresponding to linkage groups 2 and 7 in the present work. The alignment of candidate genes studied in other Rosaceous species to the BR genome assembly may provide candidate genes for chemical composition trait control in BR.

AnC in this study is the total anthocyanin content of ripe fruit measured as cyanidin 3-glucoside equivalents. Total anthocyanin is a complex BR trait consisting of multiple anthocyanin compounds [89] and influenced by many biological factors. Increased focus on distinct anthocyanin compounds, anthocyanin precursors, or anthocyanin biosynthesis pathway enzymes may provide a clearer signal for genetic analysis in a MET, as presented herein, and a more detailed understanding of anthocyanin biosynthesis and storage in BR.

## 5. Conclusions

BR is a high-value specialty crop with great market potential due to its desirable flavor and reported human health benefits. However, fresh market consumption is currently limited by the insufficient fruit quality of available cultivars arising from small fruit with highly discernable seeds. Although it was not possible to produce linkage map information for ORUS 4304, the QTL analysis performed for ORUS 4305 enabled the elucidation of genetic components contributing to fruit size and seediness, which were found to be highly heritable and fairly stable across environments. Further attention should be paid to the improvement of these traits (i.e., increased fruit size and reduced seediness), as they will be important contributors to any future success of the fresh market BR industry. Overlap between a fruit size QTL and orthologs of genes *FW2.2* and *CNR01* related to cell number regulation support the relevance of the results of this study. Genetic components contributing to fruit chemistry traits are also identified, though these components show low stability over U.S. production regions. In addition, and not least relevant, the results here yielded insights on issues in version 3 of the genome assembly for black raspberry, as well as a high incidence of progenies resulting from self-pollination in ORUS 4304. Multiple acidity and anthocyanin content QTL were identified, confirming genetic variation for these traits is available. 

## Figures and Tables

**Table 1:** Summary of variance-covariance structures fit in each model.

Matrices	Models ^a^			
	1	2	3	4
G_A×E_	CS	CS	CHS	FA(1)
R	IDV	DIAG	DIAG	DIAG

^a^ CS, compound symmetry; CHS, CS with heterogeneous variance; FA(1), factor analytic; IDV, independent with common variance; DIAG, independent with heterogeneous variance.

**Table 2:** Mixed model comparison for black raspberry fruit traits with fixed environmental effects and random additive genomic-by-environment effects (G_A×E_). G_A×E_ variance structures were fit as homogeneous with uniform correlation, heterogeneous with uniform correlation, or heterogeneous with non-uniform correlation estimated via factor analysis. Residual variance structures were fit as independent with homogeneous variance or independent with heterogeneous variance. Each model was tested against the previous model using a likelihood ratio test. FrM = fruit mass; SdM = seed mass; DrC = drupelet count; DrM = drupelet mass; SdF = seed fraction; SSC = soluble solid content; TAc = titratable acidity; AnC = anthocyanin content; PhC = phenolics content.

Trait	Model	Number of Parameters Fit	Log Likelihood	*p*-Value	
DrC	1	3	−5481.25		
DrC	2	12	−5414.73	3.65 × 10^−11^	***
DrC	3	21	−5397.96	0.026	*
DrC	4	30	−5371.33	0.001	**
DrM	1	3	−3560.17		
DrM	2	12	−3425.37	6.10 × 10^−25^	***
DrM	3	21	−3408.32	0.024	*
DrM	4	30	−3403.04	0.405	
FrM	1	3	1045.82		
FrM	2	12	1117.22	4.04 × 10^−12^	***
FrM	3	21	1138.97	0.005	**
FrM	4	30	1143.91	0.420	
SdF	1	3	−1587.19		
SdF	2	12	−1193.4	1.45 × 10^−79^	***
SdF	3	21	−1177.5	0.034	*
SdF	4	30	−1171.41	0.365	
SdM	1	3	1824.57		
SdM	2	12	1930.45	5.06 × 10^−19^	***
SdM	3	21	1948.16	0.019	*
SdM	4	30	1952.53	0.443	
TAc	1	3	2075.38		
TAc	2	13	2143.88	4.32 × 10^−11^	***
TAc	3	23	2158.62	0.071	
TAc	4	33	2163.23	0.458	
AnC	1	3	−3177.79		
AnC	2	13	−3091.47	1.43 × 10^−14^	***
AnC	3	23	−3075.18	0.046	*
AnC	4	33	−3063.74	0.162	
pH	1	3	1981.96		
pH	2	13	2018.33	3.63 × 10^−5^	***
pH	3	23	2024.84	0.385	
pH	4	33	2031.25	0.390	
PhC	1	3	−2444.53		
PhC	2	13	−2185.54	3.49 × 10^−50^	***
PhC	3	23	−2168.21	0.034	*
PhC	4	33	−2147.16	0.010	*
SSC	1	3	−1723.52		
SSC	2	13	−1484.68	6.00 × 10^−46^	***
SSC	3	23	−1458.17	0.002	**
SSC	4	33	−1451.76	0.390	

*** *p* < 0.001; ** *p* < 0.01; * *p* < 0.05.

**Table 3:** Best fitting (co)variance structures and genome-by-environment correlation estimates (*ρ*) for black raspberry fruit quality traits tested in four locations (NC, NY, OH, and OR) and three years (2013, 2014, and 2015).

Trait	Model	*ρ*	SE	*ρ*/SE
Drupelet mass	3	0.85	0.040	21.0
Fruit mass	3	0.89	0.036	25.0
Seed fraction	3	0.79	0.065	12.0
Seed mass	3	0.85	0.034	25.0
Drupelet count	4	0.88 to 1		
pH	2	0.69	0.062	11.0
Titratable acidity	2	0.79	0.046	17.0
Soluble solid content	3	0.46	0.097	4.8
Total anthocyanin content	3	0.73	0.066	11.0
Total phenolics content	4	−1 to 1		

**Table 4:** Heritability values for each trait by environment, estimated by the best-fitting model for each trait. Environments correspond to locations (NC, NY, OH, and OR) and three years (2013, 2014, and 2015).

Environment	Fruit Mass	Drupelet Count	Drupelet Mass	Seed Mass	Seed Fraction	Soluble Solid Content	Titratable Acidity	pH	Anthocyanin Content	Phenolics Content
NC 2013	0.50	0.67	0.34	0.54	0.17	0.03	0.30	0.43	0.27	0.23
NC 2014	0.28	0.38	0.43	0.39	0.46	0.13	0.33	0.37	0.37	0.01
NY 2013	0.48	0.85	0.28	0.57	0.03	0.20	0.54	0.33	0.33	0.04
NY 2014	0.41	0.66	0.34	0.61	0.02	0.23	0.41	0.42	0.42	0.49
NY 2015	0.45	0.79	0.47	0.50	0.10	0.18	0.45	0.24	0.11	0.11
OH 2013	0.70	0.83	0.73	0.70	0.45	0.29	0.53	0.49	0.46	0.26
OH 2014	0.60	0.89	0.59	0.57	0.54	0.31	0.71	0.38	0.29	0.55
OH 2015	0.54	0.84	0.69	0.60	0.43	0.21	0.55	0.39	0.28	0.12
OR 2013	na	na	na	na	na	0.02	0.46	0.34	0.20	0.04
OR 2014	0.63	0.81	0.44	0.58	0.37	0.11	0.50	0.28	0.56	0.33
OR 2015	0.49	0.81	0.55	0.69	0.63	0.05	0.50	0.28	0.35	0.43
range	0.28–0.70	0.38–0.89	0.28–0.73	0.39–0.70	0.02–0.63	0.02–0.31	0.30–0.71	0.24–0.49	0.11–0.56	0.01–0.55

**Table 5:** Summary of QTL identified by single trait, multiple-environment analysis. FrM = fruit mass; SdM = seed mass; DrC = drupelet count; DrM = drupelet mass; SdF = seed fraction; SSC = soluble solid content; TAc = titratable acidity; AnC = anthocyanin content; PhC = phenolics content.

Trait	Number of QTL	QTL Name	LG	Position	Marker Name	−log_10_(*p*)	QTL × E
FrM	4	qRoc-FrM1.1	1	41.7	SRO01_5349433	9.17	N
		qRoc-FrM1.2	1	106.5	SRO01_23458156	14.00	Y
		qRoc-FrM2.1	2	0	SRO02_329222	3.45	Y
		qRoc-FrM6.1	6	34.4	SRO06_21156325	8.64	N
SdM	0						
DrC	3	qRoc-DrC1.1	1	41.7	SRO01_5349433	42.94	N
		qRoc-DrC1.2	1	104.6	SRO01_23058326	16.24	Y
		qRoc-DrC4.1	4	3.2	SRO04_451654	7.93	Y
DrM	3	qRoc-DrM1.1	1	43.2	SRO01_6243993	10.73	N
		qRoc-DrM1.2	1	93.31	SRO01_23565201	1.40	N
		qRoc-DrM6.1	6	15.38	SRO06_185350	7.58	Y
SdF	3	qRoc-SdF2.1	2	46.6	SRO02_3650689	11.59	Y
		qRoc-SdF6.1	6	16.0	SRO06_251781	11.67	N
		qRoc-SdF7.1	7	120.4	SRO07_28298811	6.96	Y
SSC	0						
TAc	3	qRoc-TAc3.1	3	6.6	Ri11795_SSR	6.06	Y
		qRoc-TAc4.1	4	70.6	Ro_CBEa0004G23	3.78	Y
		qRoc-TAc6.1	6	78.3	SRO06_13350974	3.93	Y
AnC	1	qRoc-AnC3.1	3	163.5	Ro17045_SSR	6.02	Y
PhC	0						

**Table 6:** Titratable acidity QTL identified by single-environment analysis. Single-environment analysis was performed using the R software ‘qtl’ package. Best-fit additive models were selected via forward/backward selection. Penalty values were calculated from 1000 permutations of a two-dimensional, two-QTL scan (*p* ≤ 0.1).

Env	Model	QTL	Chr	Pos	LOD
NC 2013	null				
NC 2014	y~Q1	Q1	1	104.6	3.9
NY 2013	y~Q1	Q1	3	9.3	4.5
NY 2014	null				
NY 2015	null				
OH 2013	y~Q1+Q2+Q3	Q1	1	106.0	6.5
	y~Q1+Q2+Q3	Q2	4	114.0	3.8
	y~Q1+Q2+Q3	Q3	4	121.3	7.3
OH 2014	null				
OH 2015	y~Q1+Q2+Q3	Q1	3	19.8	4.0
	y~Q1+Q2+Q3	Q2	4	56.8	5.9
	y~Q1+Q2+Q3	Q3	6	51.2	5.2
OR 2013	y~Q1	Q1	6	55.4	5.3
OR 2014	null				
OR 2015	y~Q1	Q1	4	119.2	6.8

## Data Availability

The online version of this article contains Supplementary Materials, which is available to authorized users. Sequencing data can be made available upon request.

## Supplementary Materials

The following are available online at https://www.mdpi.com/article/10.3390/genes13030418/s1. File 1: Supplementary_File_1; File 2: Supplementary_File_2, Figure S1: Distributions of average fruit mass observed in ten location-by-year environments, Figure S2: Distributions of average seed mass observed in ten location-by-year environments, Figure S3: Distributions of average drupelet count observed in ten location-by-year environments, Figure S4: Distributions of seed fraction observed in ten location-by-year environments. Seed fraction was calculated as total seed mass/total fruit mass × 100%, Figure S5: Distributions of drupelet mass observed in ten location-by-year environments, Figure S6: Distributions of soluble solid content observed in eleven location-by-year environments, Figure S7: Distributions of titratable acidity observed in eleven location-by-year environments, Figure S8: Distributions of pH in eleven location-by-year environments, Figure S9: Distributions of anthocyanin content observed in eleven location-by-year environments. C3G = cyanadin 3-glucoside, Figure S10: Distributions of phenolics content observed in eleven location-by-year environments. GAE = gallic acid equivalents, Table S1: Number of ORUS 4305 individuals with phenotypic data for fruit size (FrM, SdM, DrC, SdF) and fruit chemistry (TAc, AnC, PhC) within trial environments.

## References

[1] Bradish C.M., Fernandez G.E., Bushakra J.M., Perkins-Veazie P., Dossett M., Bassil N.V., Finn C.E. (2016). Evaluation of Vigor and Winter Hardiness of Black Raspberry Breeding Populations (*Rubus occidentalis*) Grown in the Southeastern US. Acta Hortic..

[2] Jennings D.L. (1988). Raspberries and Blackberries: Their Breeding, Diseases and Growth.

[3] Daubeny H.A., Janick J., Moore J.N. (1996). Brambles. Fruit Breeding.

[4] Slate G.L., Klein L.G. (1952). Black Raspberry Breeding. Proc. Am. Soc. Hort. Sci..

[5] Drain B.D. (1953). Some Inheritance Data with Black Raspberries. Proc. Am. Soc. Hort. Sci..

[6] Drain B.D. (1956). Inheritance in Black Raspberry Species. Proc. Am. Soc. Hort. Sci..

[7] Weber C.A. (2003). Genetic Diversity in Black Raspberry Detected by RAPD Markers. HortScience.

[8] Dossett M., Bassil N.V., Lewers K.S., Finn C.E. (2012). Genetic Diversity in Wild and Cultivated Black Raspberry (*Rubus occidentalis* L.) Evaluated by Simple Sequence Repeat Markers. Genet. Resour. Crop Evol..

[9] Amiot M.J., Riva C., Vinet A. (2016). Effects of Dietary Polyphenols on Metabolic Syndrome Features in Humans: A Systematic Review. Obes. Rev..

[10] Kresty L.A., Mallery S.R., Stoner G.D. (2016). Black Raspberries in Cancer Clinical Trials: Past, Present and Future. J. Berry Res..

[11] USDA (2017). National Statistics for Raspberries.

[12] Dossett M., Lee J., Finn C.E. (2008). Inheritance of Phenological, Vegetative, and Fruit Chemistry Traits in Black Raspberry. J. Am. Soc. Hortic. Sci..

[13] Fang J. (2015). Classification of Fruits Based on Anthocyanin Types and Relevance to Their Health Effects. Nutrition.

[14] Dossett M., Lee J., Finn C.E. (2010). Variation in Anthocyanins and Total Phenolics of Black Raspberry Populations. J. Funct. Foods.

[15] Dossett M., Lee J., Finn C.E. (2012). Anthocyanin Content of Wild Black Raspberry Germplasm. Acta Hortic..

[16] Dossett M. (2011). Evaluation of Genetic Diversity of Wild Populations of Black Raspberry (*Rubus occidentalis* L.). Ph.D. Dissertation.

[17] Ozgen M., Wyzgoski F.J., Tulio A.Z., Gazula A., Miller A.R., Scheerens J.C., Reese R.N., Wright S.R. (2008). Antioxidant Capacity and Phenolic Antioxidants of Midwestern Black Raspberries Grown for Direct Markets Are Influenced by Production Site. HortScience.

[18] Perkins-Veazie P., Ma G., Fernandez G.E., Bradish C.M., Bushakra J.M., Bassil N.V., Weber C.A., Scheerens J.C., Robbins L., Finn C.E. (2016). Black Raspberry Fruit Composition over Two Years from Seedling Populations Grown at Four US Geographic Locations. Acta Hortic..

[19] Bushakra J.M., Bradish C.M., Weber C.A., Dossett M., Fernandez G., Weiland J., Peterson M., Scheerens J.C., Robbins L., Serçe S. (2016). Toward Understanding Genotype × Environment Interactions in Black Raspberry (*Rubus occidentalis* L.). Proceedings of the XXIX International Horticultural Congress on Horticulture: Sustaining Lives, Livelihoods and Landscapes (IHC2014): II International Berry Fruit Symposium: Interactions! Local and Global Berry Research and Innovation.

[20] Dossett M., Finn C.E. (2010). Identification of Resistance to the Large Raspberry Aphid in Black Raspberry. J. Am. Soc. Hortic. Sci..

[21] Finn C., Knight V.H. (2002). What’s Going on in the World of *Rubus* Breeding?. Acta Hortic..

[22] Bushakra J.M., Bryant D.W., Dossett M., Vining K.J., VanBuren R., Gilmore B.S., Lee J., Mockler T.C., Finn C.E., Bassil N.V. (2015). A Genetic Linkage Map of Black Raspberry (*Rubus occidentalis*) and the Mapping of *Ag 4* Conferring Resistance to the Aphid *Amphorophora agathonica*. Theor. Appl. Genet..

[23] Bushakra J.M., Dossett M., Carter K.A., Vining K.J., Lee J.C., Bryant D.W., VanBuren R., Lee J., Mockler T.C., Finn C.E. (2018). Characterization of Aphid Resistance Loci in Black Raspberry (*Rubus occidentalis* L.). Mol. Breed..

[24] Byrne D.H. (2012). Trends in Fruit Breeding. Fruit Breeding, Handbook of Plant Breeding.

[25] van Eeuwijk F.A. (1995). Linear and Bilinear Models for the Analysis of Multi-Environment Trials: I. An Inventory of Models. Euphytica.

[26] Smith A.B., Cullis B.R., Thompson R. (2005). The Analysis of Crop Cultivar Breeding and Evaluation Trials: An Overview of Current Mixed Model Approaches. J. Agric. Sci..

[27] Hardner C., Winks C., Stephenson R., Gallagher E. (2001). Genetic Parameters for Nut and Kernel Traits in Macadamia. Euphytica.

[28] Hardner C.M., Hayes B.J., Kumar S., Vanderzande S., Cai L., Piaskowski J., Quero-Garcia J., Campoy J.A., Barreneche T., Giovannini D. (2019). Prediction of Genetic Value for Sweet Cherry Fruit Maturity among Environments Using a 6K SNP Array. Hortic. Res..

[29] Hardner C. (2017). Exploring Opportunities for Reducing Complexity of Genotype-by-Environment Interaction Models. Euphytica.

[30] Smith A.B., Cullis B.R. (2018). Plant Breeding Selection Tools Built on Factor Analytic Mixed Models for Multi-Environment Trial Data. Euphytica.

[31] Boer M.P., Wright D., Feng L., Podlich D.W., Luo L., Cooper M., van Eeuwijk F.A. (2007). A Mixed-Model Quantitative Trait Loci (QTL) Analysis for Multiple-Environment Trial Data Using Environmental Covariables for QTL-by-Environment Interactions, with an Example in Maize. Genetics.

[32] Malosetti M., Ribaut J.M., Vargas M., Crossa J., van Eeuwijk F.A. (2008). A Multi-Trait Multi-Environment QTL Mixed Model with an Application to Drought and Nitrogen Stress Trials in Maize (*Zea mays* L.). Euphytica.

[33] Ourecky D.K., Slate G.L. (1973). Jewel Black Raspberry. N. Y. Food Life Sci. Bull..

[34] Lodhi M.A., Ye G.-N., Weeden N.F., Reisch B.I. (1994). A Simple and Efficient Method for DNA Extraction from Grapevine Cultivars and *Vitis* Species. Plant Mol. Biol. Rep..

[35] Elshire R.J., Glaubitz J.C., Sun Q., Poland J.A., Kawamoto K., Buckler E.S., Mitchell S.E. (2011). A Robust, Simple Genotyping-by-Sequencing (GBS) Approach for High Diversity Species. PLoS ONE.

[36] Andrews S. (2010). FastQC: A Quality Control Tool for High Throughput Sequence Data. http://www.Bioinformatics.Babraham.Ac.Uk/Projects/Fastqc.

[37] Bolger A.M., Lohse M., Usadel B. (2014). Trimmomatic: A Flexible Trimmer for Illumina Sequence Data. Bioinformatics.

[38] Glaubitz J.C., Casstevens T.M., Lu F., Harriman J., Elshire R.J., Sun Q., Buckler E.S. (2014). TASSEL-GBS: A High Capacity Genotyping by Sequencing Analysis Pipeline. PLoS ONE.

[39] VanBuren R., Wai C.M., Colle M., Wang J., Sullivan S., Bushakra J.M., Liachko I., Vining K.J., Dossett M., Finn C.E. (2018). A near Complete, Chromosome-Scale Assembly of the Black Raspberry (*Rubus occidentalis*) Genome. GigaScience.

[40] Danecek P., Auton A., Abecasis G., Albers C.A., Banks E., DePristo M.A., Handsaker R.E., Lunter G., Marth G.T., Sherry S.T. (2011). The Variant Call Format and VCFtools. Bioinformatics.

[41] Yang J., Benyamin B., McEvoy B.P., Gordon S., Henders A.K., Nyholt D.R., Madden P.A., Heath A.C., Martin N.G., Montgomery G.W. (2010). Common SNPs Explain a Large Proportion of the Heritability for Human Height. Nat. Genet..

[42] R Core Team (2018). R: A Language and Environment for Statistical Computing.

[43] Gilmour A.R., Gogel B., Cullis B.R., Thompson R., Welham S.J. (2015). ASReml User Guide Release 4.1.

[44] Wimmer V., Albrecht T., Auinger H.-J., Schön C.-C. (2012). Synbreed: A Framework for the Analysis of Genomic Prediction Data Using R. Bioinformatics.

[45] VanRaden P.M. (2008). Efficient Methods to Compute Genomic Predictions. J. Dairy Sci..

[46] Isik F., Holland J., Maltecca C. (2017). Multienvironmental Trials. Genetic Data Analysis for Plant and Animal Breeding.

[47] Van Ooijen J.W., Jansen J. (2013). Genetic Mapping in Experimental Populations.

[48] Van Ooijen J.W. (2006). JoinMap^®^ 4.0: Software for the Calculation of Genetic Linkage Maps in Experimental Populations.

[49] Van Ooijen J.W. (2011). Multipoint Maximum Likelihood Mapping in a Full-Sib Family of an Outbreeding Species. Genet. Res..

[50] VSN International (2018). GenStat for Windows.

[51] Li J., Ji L. (2005). Adjusting Multiple Testing in Multilocus Analyses Using the Eigenvalues of a Correlation Matrix. Heredity.

[52] Broman K.W., Wu H., Sen S., Churchill G.A. (2003). R/Qtl: QTL Mapping in Experimental Crosses. Bioinformatics.

[53] Voorrips R.E. (2002). MapChart: Software for the Graphical Presentation of Linkage Maps and QTLs. J. Hered..

[54] Sanford J.C., Ourecky D.K., Reich J.E. (1985). “Titan” Red Raspberry. HortScience.

[55] Frary A. (2000). Fw2.2: A Quantitative Trait Locus Key to the Evolution of Tomato Fruit Size. Science.

[56] Guo M., Rupe M.A., Dieter J.A., Zou J., Spielbauer D., Duncan K.E., Howard R.J., Hou Z., Simmons C.R. (2010). Cell Number Regulator1 Affects Plant and Organ Size in Maize: Implications for Crop Yield Enhancement and Heterosis. Plant Cell.

[57] De Franceschi P., Stegmeir T., Cabrera A., van der Knaap E., Rosyara U.R., Sebolt A.M., Dondini L., Dirlewanger E., Quero-Garcia J., Campoy J.A. (2013). Cell Number Regulator Genes in *Prunus* Provide Candidate Genes for the Control of Fruit Size in Sweet and Sour Cherry. Mol. Breed..

[58] Karaagac E., Vargas A., Andrés M., Carreño I., Ibáñez J., Carreño J., Martínez-Zapater J., Cabezas J. (2012). Marker Assisted Selection for Seedlessness in Table Grape Breeding. Tree Genet. Genomes.

[59] Royo C., Torres-Pérez R., Mauri N., Diestro N., Cabezas J.A., Marchal C., Lacombe T., Ibáñez J., Tornel M., Carreño J. (2018). The Major Origin of Seedless Grapes Is Associated with a Missense Mutation in the MADS-Box Gene *VviAGL11*. Plant Physiol..

[60] Vardi A., Levin I., Carmi N. (2008). Induction of Seedlessness in Citrus: From Classical Techniques to Emerging Biotechnological Approaches. J. Am. Soc. Hortic. Sci..

[61] Guo Y., Gao M., Liang X., Xu M., Liu X., Zhang Y., Liu X., Liu J., Gao Y., Qu S. (2020). Quantitative Trait Loci for Seed Size Variation in Cucurbits—A Review. Front. Plant Sci..

[62] Card F.W. (1898). Bush-Fruits.

[63] Darrow G.M., Sherwood H. (1931). Seed and Berry Size of Cane Fruits. Proc. Am. Soc. Hort. Sci..

[64] Sebesta B., Clark J.R., Threlfall R.T., Howard L.R. (2013). Characterization of Seediness Attributes of Blackberry Genotypes. Discov. Stud. J. Dale Bump. Coll. Agric. Food Life Sci..

[65] Hummer K.E., Peacock D.N. (1994). Seed Dimension and Weight of Selected *Rubus* Species. HortScience.

[66] Hancock R.D., Petridis A., McDougall G.J. (2018). Raspberry Fruit Chemistry in Relation to Fruit Quality and Human Nutrition. Raspberries: Breeding, Challenges, and Advances.

[67] Mazur S.P., Nes A., Wold A.-B., Remberg S.F., Aaby K. (2014). Quality and Chemical Composition of Ten Red Raspberry (*Rubus idaeus* L.) Genotypes during Three Harvest Seasons. Food Chem..

[68] Mazur S.P., Sønsteby A., Wold A.-B., Foito A., Freitag S., Verrall S., Conner S., Stewart D., Heide O.M. (2014). Post-Flowering Photoperiod Has Marked Effects on Fruit Chemical Composition in Red Raspberry (*Rubus idaeus*). Ann. Appl. Biol..

[69] Graham J., Brennan R. (2018). Raspberries: Breeding, Challenges, and Advances.

[70] Singleton V.L., Orthofer R., Lamuela-Raventos R.M. (1999). Analysis of Total Phenolics and Other Oxidation Substrates and Antioxidants by Means of Folin-Ciocalteu Reagent. Methods Enzymol..

[71] Klee H.J. (2010). Improving the Flavor of Fresh Fruits: Genomics, Biochemistry, and Biotechnology: Tansley Review. New Phytol..

[72] Khoo H.E., Azlan A., Tang S.T., Lim S.M. (2017). Anthocyanidins and Anthocyanins: Colored Pigments as Food, Pharmaceutical Ingredients, and the Potential Health Benefits. Food Nutr. Res..

[73] Etienne A., Génard M., Lobit P., Mbeguié-A-Mbéguié D., Bugaud C. (2013). What Controls Fleshy Fruit Acidity? A Review of Malate and Citrate Accumulation in Fruit Cells. J. Exp. Biol..

[74] Malowicki S.M.M., Martin R., Qian M.C. (2008). Comparison of Sugar, Acids, and Volatile Composition in Raspberry Bushy Dwarf Virus-Resistant Transgenic Raspberries and the Wild Type ‘Meeker’ (*Rubus idaeus* L.). J. Agric. Food Chem..

[75] Vool E., Karp K., Noormets M., Moor U., Starast M. (2009). The Productivity and Fruit Quality of the Arctic Bramble (*Rubus Arcticus* Ssp. *Arcticus*) and Hybrid Arctic Bramble (*Rubus arcticus* asp. *arcticus* × *Rubus arcticus* ssp. *stellatus*). Acta Agric. Scand. Sect. B Soil Plant Sci..

[76] Etienne C., Rothan C., Moing A., Plomion C., Bodénès C., Svanella-Dumas L., Cosson P., Pronier V., Monet R., Dirlewanger E. (2002). Candidate Genes and QTLs for Sugar and Organic Acid Content in Peach [*Prunus Persica* (L.) Batsch]. Theor. Appl. Genet..

[77] Krüger E., Dietrich H., Schöpplein E., Rasim S., Kürbel P. (2011). Cultivar, Storage Conditions and Ripening Effects on Physical and Chemical Qualities of Red Raspberry Fruit. Postharvest Biol. Technol..

[78] Lee J., Dossett M., Finn C.E. (2012). *Rubus* Fruit Phenolic Research: The Good, the Bad, and the Confusing. Food Chem..

[79] Mazzoni L., Perez-Lopez P., Giampieri F., Alvarez-Suarez J.M., Gasparrini M., Forbes-Hernandez T.Y., Quiles J.L., Mezzetti B., Battino M. (2016). The Genetic Aspects of Berries: From Field to Health: The Genetic Aspects of Berries. J. Sci. Food Agric..

[80] Chaves-Silva S., dos Santos A.L., Chalfun-Júnior A., Zhao J., Peres L.E.P., Benedito V.A. (2018). Understanding the Genetic Regulation of Anthocyanin Biosynthesis in Plants—Tools for Breeding Purple Varieties of Fruits and Vegetables. Phytochemistry.

[81] Scalzo J., Battino M., Costantini E., Mezzetti B. (2005). Breeding and Biotechnology for Improving Berry Nutritional Quality. Biofactors.

[82] Allan A.C., Hellens R.P., Laing W.A. (2008). MYB Transcription Factors That Colour Our Fruit. Trends Plant Sci..

[83] Griesser M., Hoffmann T., Bellido M.L., Rosati C., Fink B., Kurtzer R., Aharoni A., Munoz-Blanco J., Schwab W. (2008). Redirection of Flavonoid Biosynthesis through the Down-Regulation of an Anthocyanidin Glucosyltransferase in Ripening Strawberry Fruit. Plant Physiol..

[84] Zorrilla-Fontanesi Y., Cabeza A., Domínguez P., Medina J.J., Valpuesta V., Denoyes-Rothan B., Sánchez-Sevilla J.F., Amaya I. (2011). Quantitative Trait Loci and Underlying Candidate Genes Controlling Agronomical and Fruit Quality Traits in Octoploid Strawberry (Fragaria × Ananassa). Theor. Appl. Genet..

[85] Shulaev V., Sargent D.J., Crowhurst R.N., Mockler T.C., Folkerts O., Delcher A.L., Jaiswal P., Mockaitis K., Liston A., Mane S.P. (2011). The Genome of Woodland Strawberry (*Fragaria vesca*). Nat. Genet..

[86] Teng H., Fang T., Lin Q., Song H., Liu B., Chen L. (2017). Red Raspberry and Its Anthocyanins: Bioactivity beyond Antioxidant Capacity. Trends Food Sci. Technol..

[87] Hyun T.K., Lee S., Rim Y., Kumar R., Han X., Lee S.Y., Lee C.H., Kim J.-Y. (2014). De-Novo RNA Sequencing and Metabolite Profiling to Identify Genes Involved in Anthocyanin Biosynthesis in Korean Black Raspberry (*Rubus coreanus* Miquel). PLoS ONE.

[88] Kassim A., Poette J., Paterson A., Zait D., McCallum S., Woodhead M., Smith K., Hackett C., Graham J. (2009). Environmental and Seasonal Influences on Red Raspberry Anthocyanin Antioxidant Contents and Identification of Quantitative Traits Loci (QTL). Mol. Nutr. Food Res..

[89] Paudel L., Wyzgoski F.J., Giusti M.M., Johnson J.L., Rinaldi P.L., Scheerens J.C., Chanon A.M., Bomser J.A., Miller A.R., Hardy J.K. (2014). NMR-Based Metabolomic Investigation of Bioactivity of Chemical Constituents in Black Raspberry (*Rubus occidentalis* L.) Fruit Extracts. J. Agric. Food Chem..

