# Supplementary material for: Analysis of a Multi-Environment Trial for Black Raspberry (Rubus occidentalis L.) Quality Traits
Source: Genes (Basel). 2022 Feb 25;13(3):418. doi: 10.3390/genes13030418 (PMC8950803; doi:10.3390/genes13030418)
Supplement: Supplementary file 1 [file genes-13-00418-s001.zip › Supplementary_File_2.pdf]

Analysis of a multi-environment trial for black raspberry (*Rubus occidentalis* L.) quality traits

Matthew R. Willman [1], Jill M. Bushakra [2], Nahla Bassil [2], Chad. E. Finn [3,+], Michael Dossett [4], Penelope Perkins-Veazie [5], Christine M. Bradish [5], Gina E. Fernandez [5], Courtney A. Weber [6], Joseph C. Scheerens [1], Lisa Dunlap [1] and Jonathan Fresnedo-Ramírez[1,*]

1 Department of Horticulture and Crop Science, The Ohio State University, Wooster, Ohio, USA.
2 USDA-ARS, National Clonal Germplasm Repository, Corvallis, Oregon, USA.
3 USDA-ARS, Horticultural Crops Research Unit, Corvallis, Oregon, USA.
4 BC Berry Cultivar Development Inc., Abbotsford, BC, Canada.
5 Department of Horticultural Science, North Carolina State University, Raleigh, North Carolina, USA.
6 Cornell AgriTech, New York State Agricultural Experiment Station, Geneva, New York, USA.
+ Deceased.
* Correspondence: J. Fresnedo-Ramírez, Department of Horticulture and Crop Science, The Ohio State University, 1680 Madison Ave. Wooster, Ohio, 44691, USA., email: fresnedoramirez.1@osu.edu, ORCID: 0000-0003-1985-2513

Supplementary File 2:

Figure S1. Distributions of average fruit mass observed in ten location-by-year environments.
Figure S2. Distributions of average seed mass observed in ten location-by-year environments
Figure S3. Distributions of average drupelet count observed in ten location-by-year environments.
Figure S4. Distributions of seed fraction observed in ten location-by-year environments. Seed fraction was calculated as total seed mass / total fruit mass × 100%.
Figure S5. Distributions of drupelet mass observed in ten location-by-year environments.
Figure S6. Distributions of soluble solid content observed in eleven location-by-year environments.
Figure S7. Distributions of titratable acidity observed in eleven location-by-year environments.
Figure S8. Distributions of pH in eleven location-by-year environments.
Figure S9. Distributions of anthocyanin content observed in eleven location-by-year environments. C3G=cyanadin 3-glucoside.
Figure S10. Distributions of phenolics content observed in eleven location-by-year environments. GAE=gallic acid equivalents.

Table S1. Number of ORUS 4305 individuals with phenotypic data for fruit size (FrM, SdM, DrC, SdF) and fruit chemistry (TAc, AnC, PhC) within trial environments.

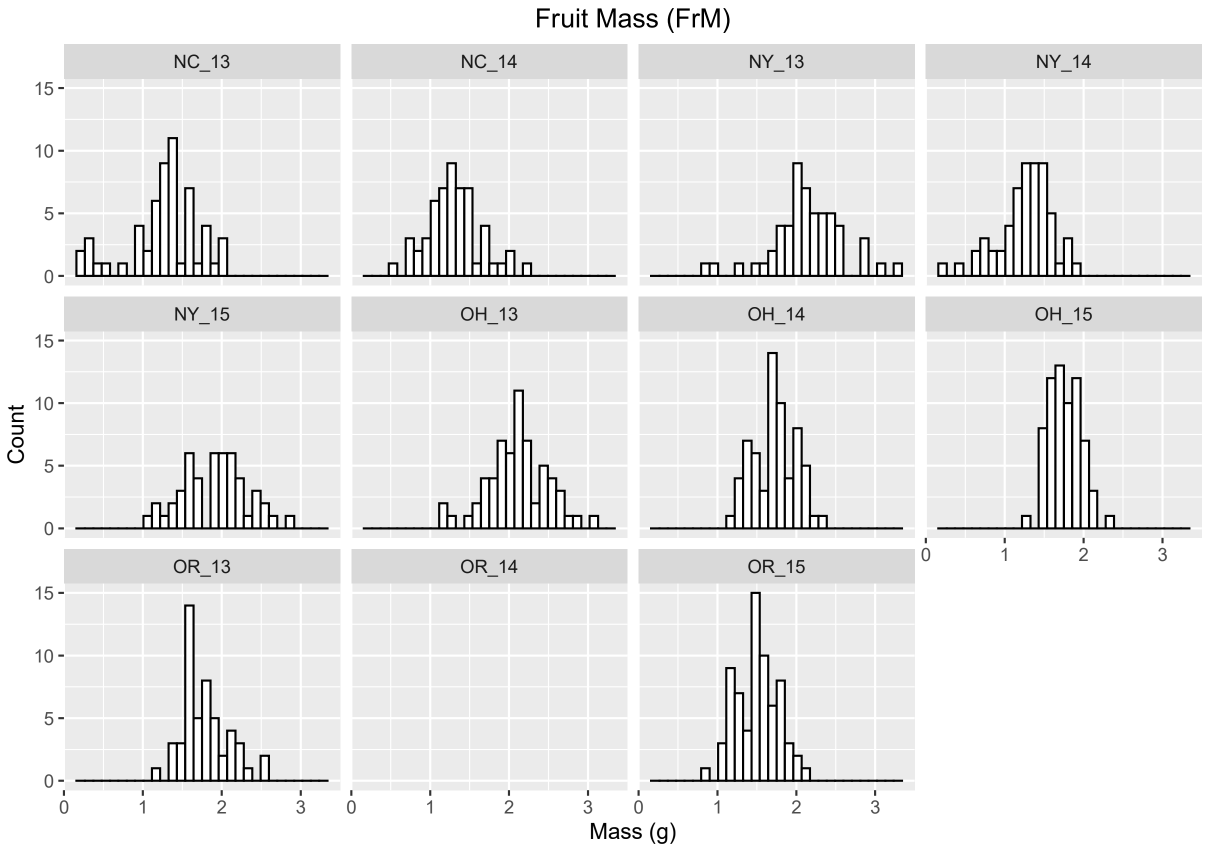

Figure S1. Distributions of average fruit mass observed in ten location-by-year environments.

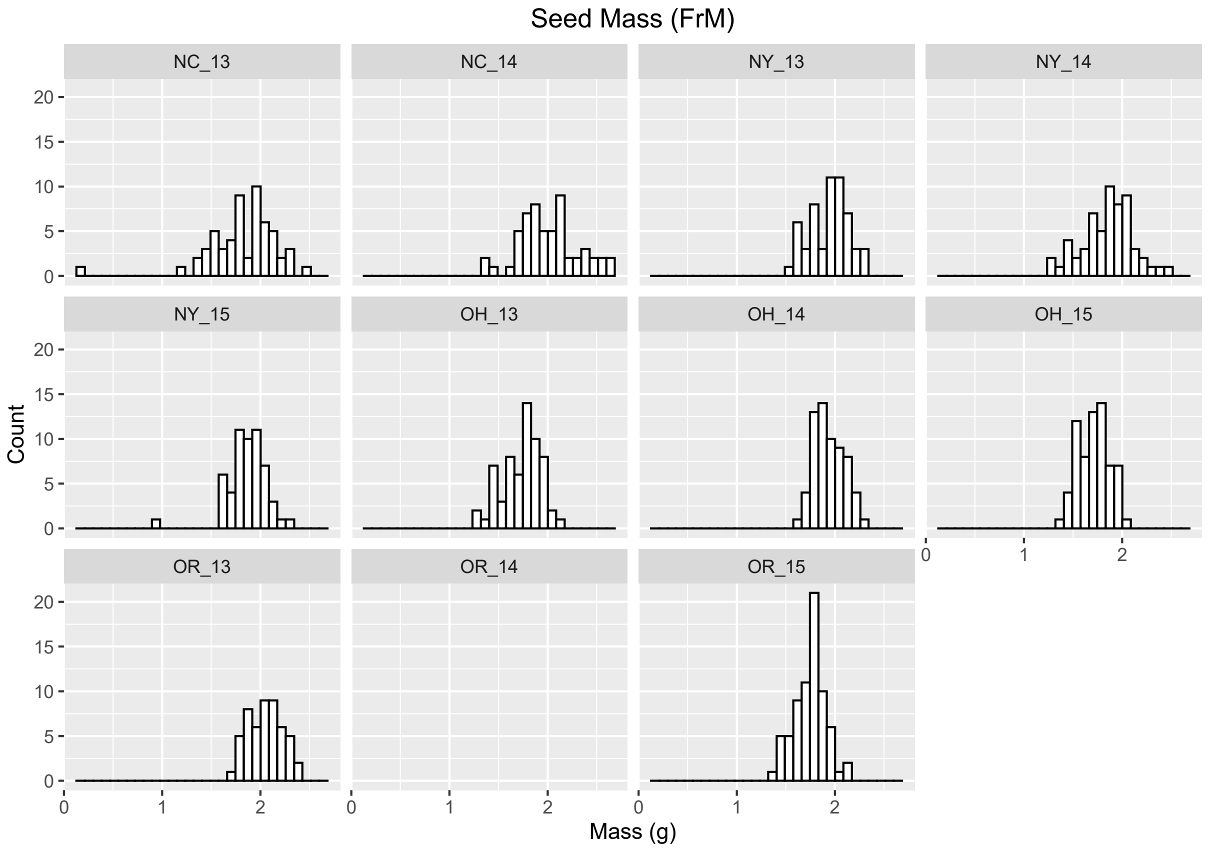

Figure S2. Distributions of average seed mass observed in ten location-by-year environments.

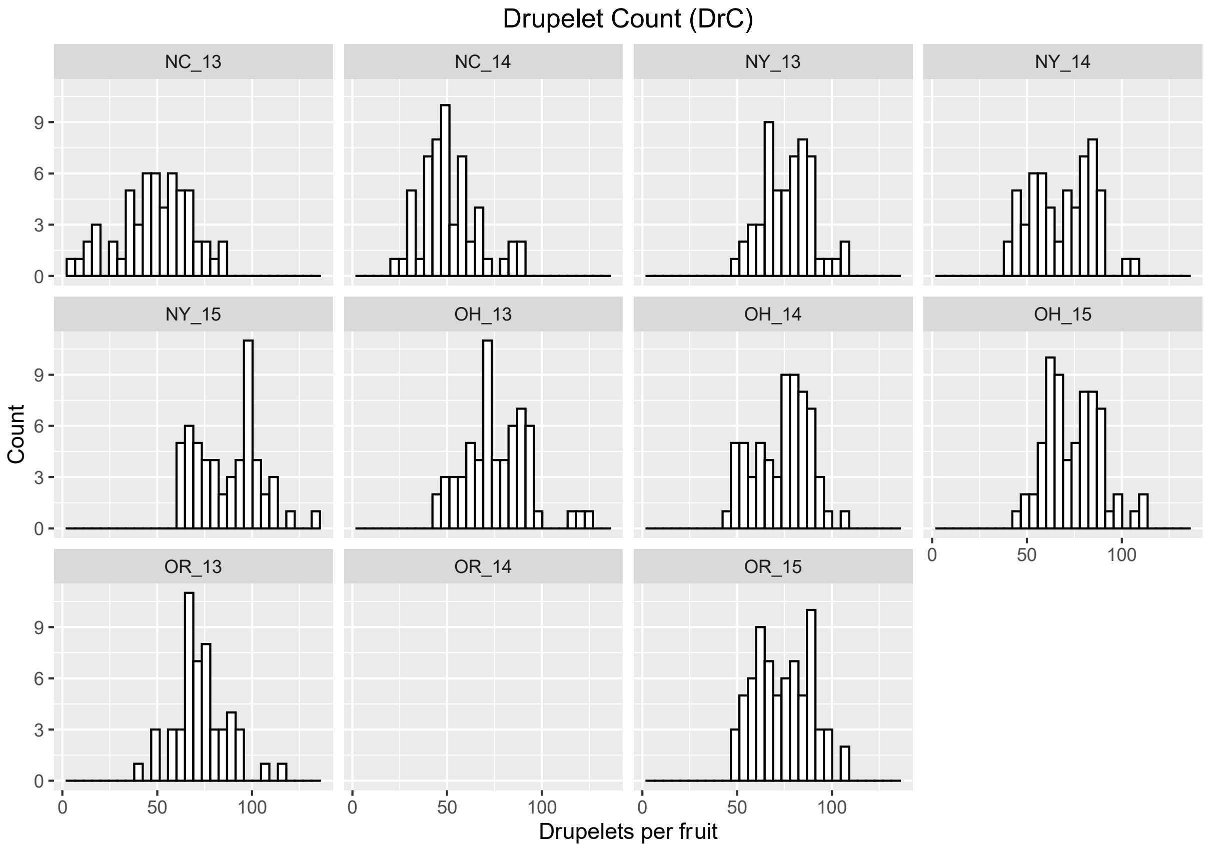

Figure S3. Distributions of average drupelet count observed in ten location-by-year environments.

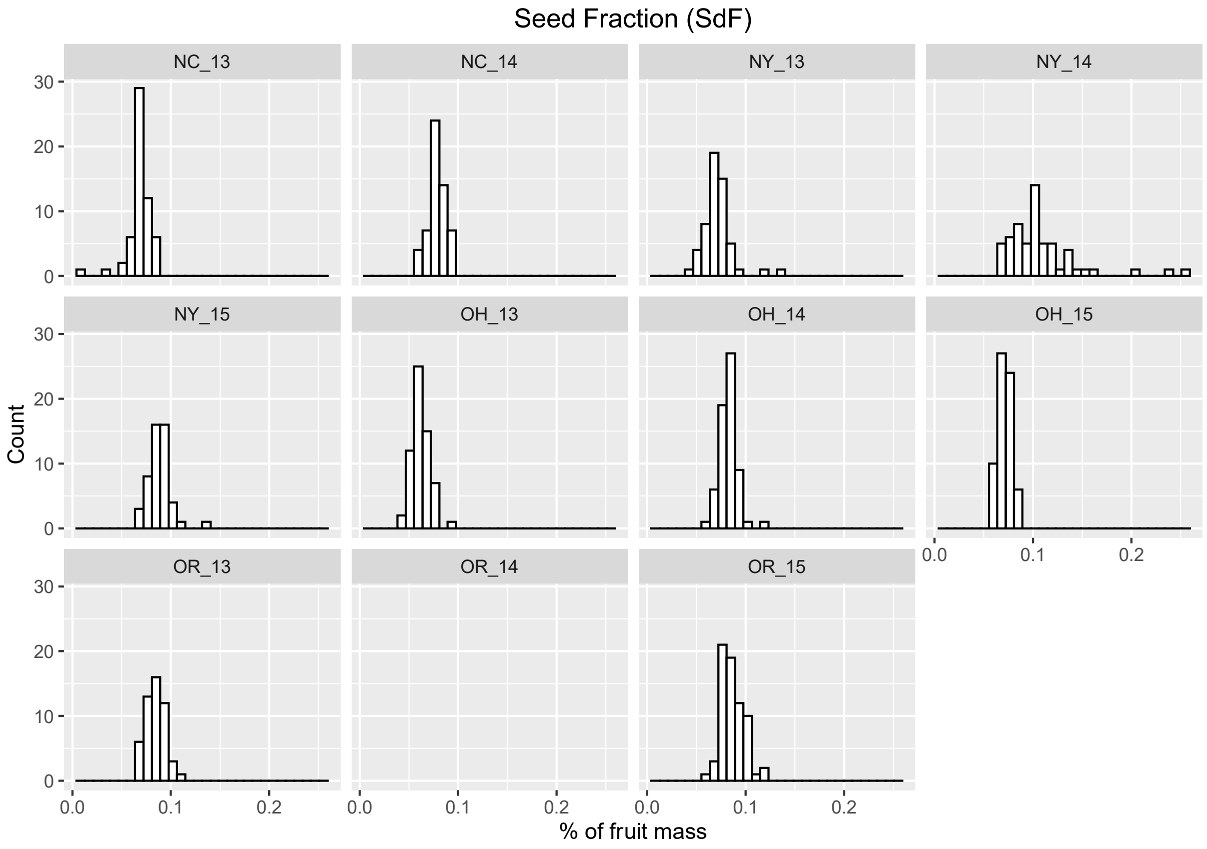

Figure S4. Distributions of seed fraction observed in ten location-by-year environments. Seed fraction was calculated as total seed mass / total fruit mass × 100%.

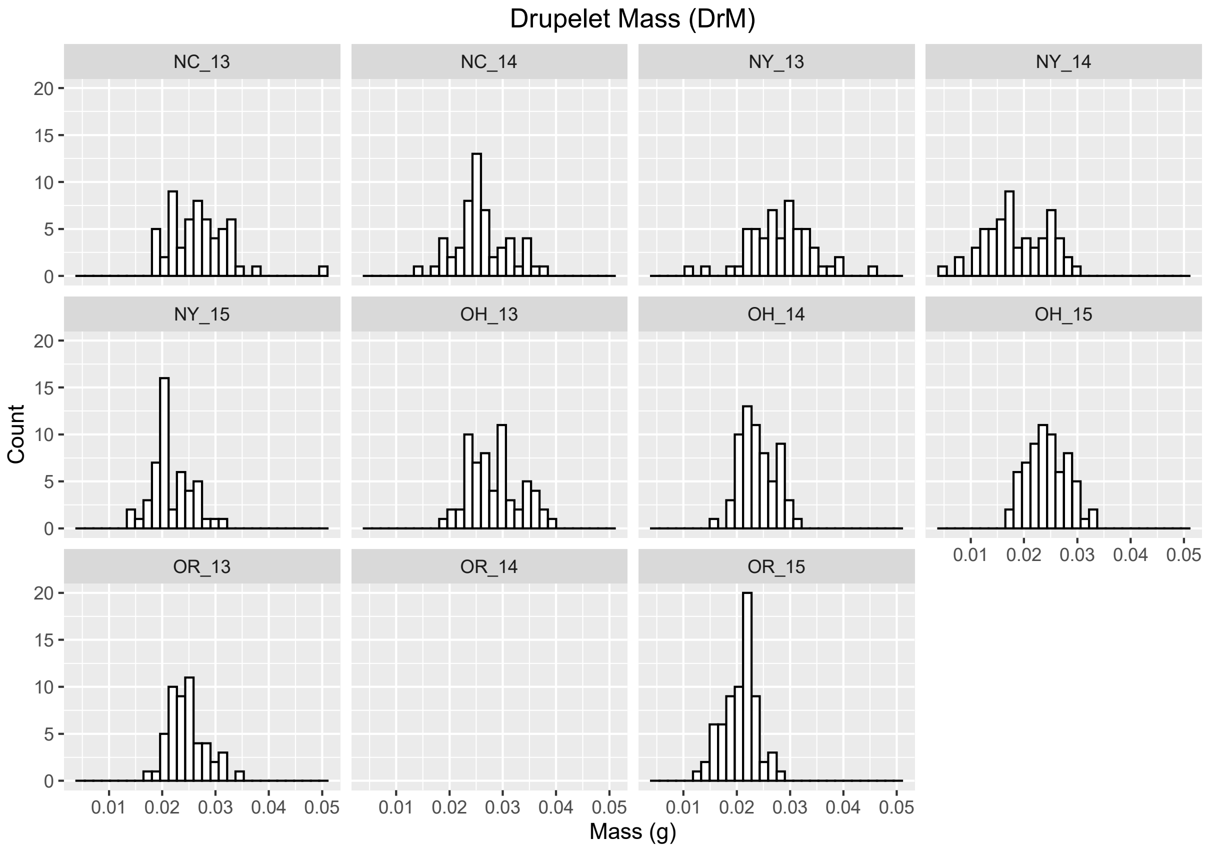

Figure S5. Distributions of drupelet mass observed in ten location-by-year environments.

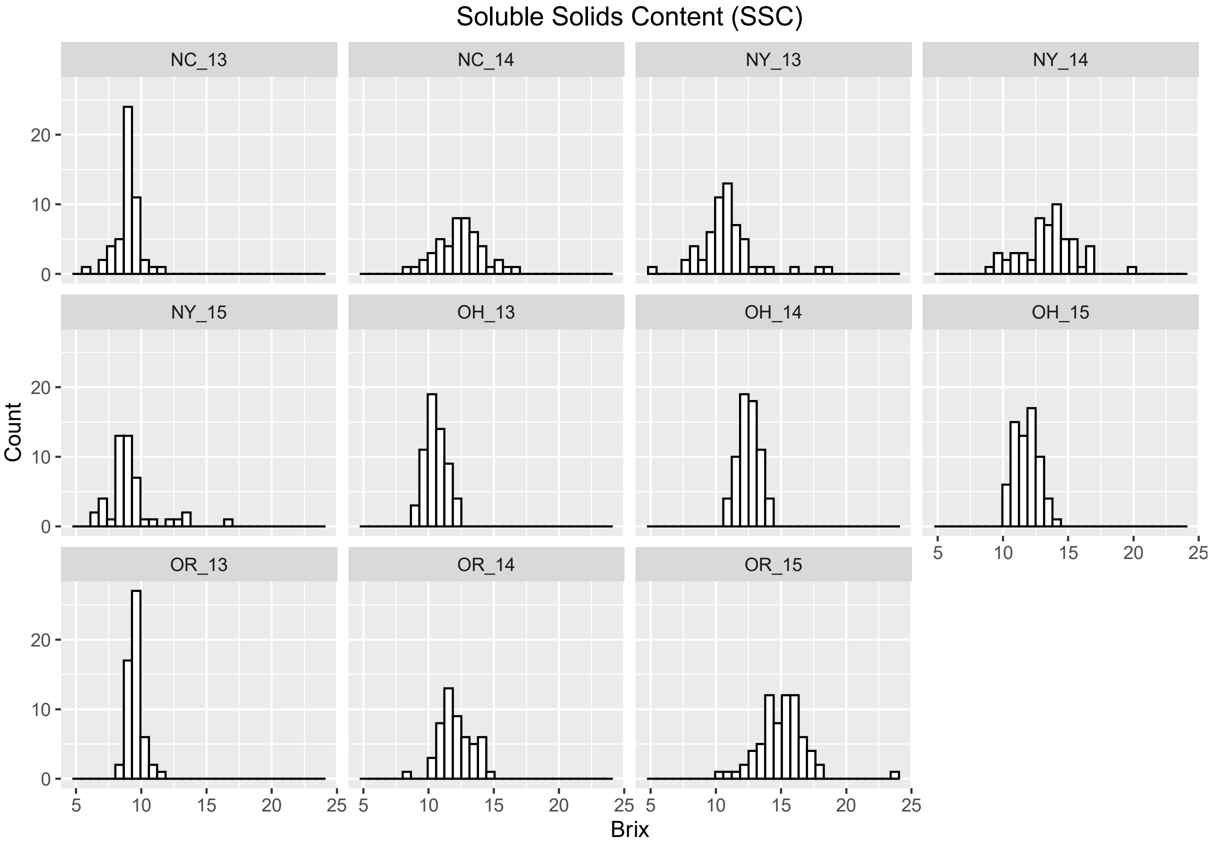

Figure S6. Distributions of soluble solid content observed in eleven location-by-year environments.

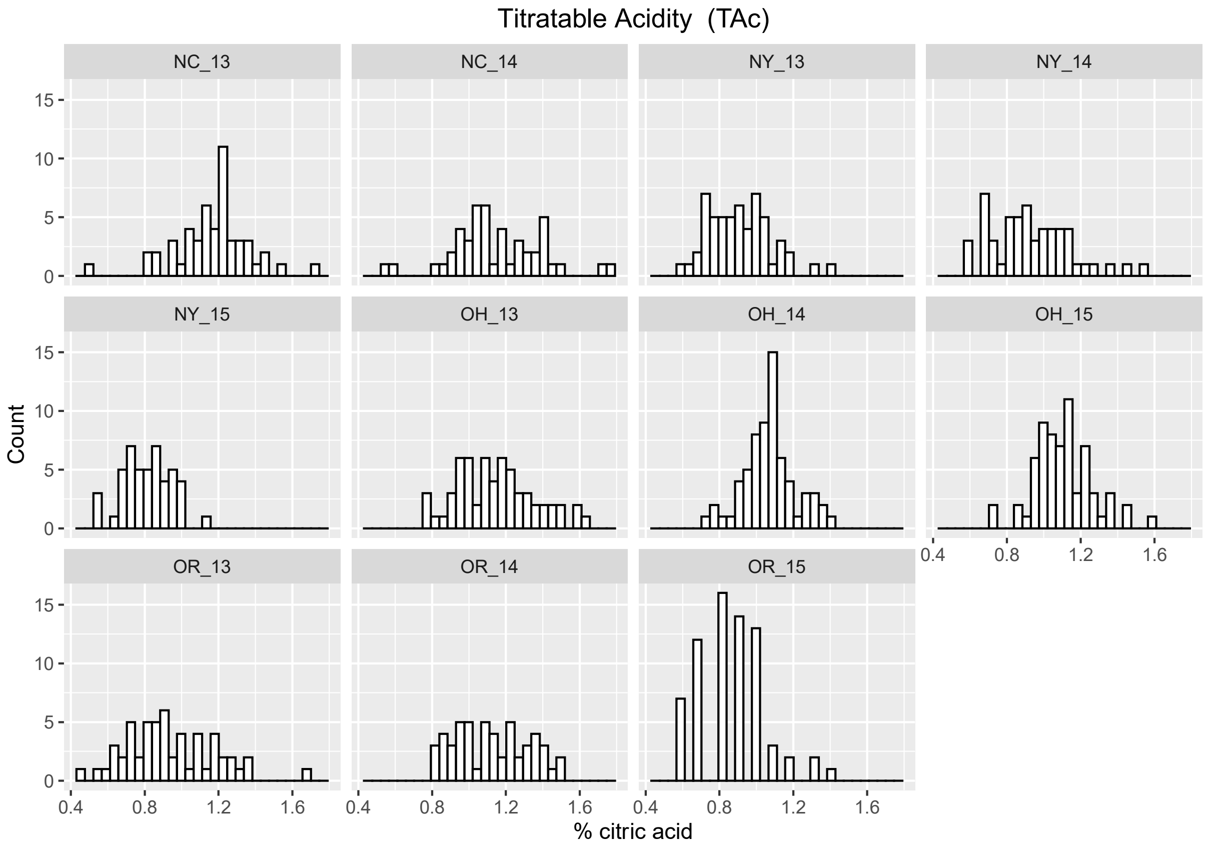

Figure S7. Distributions of titratable acidity observed in eleven location-by-year environments.

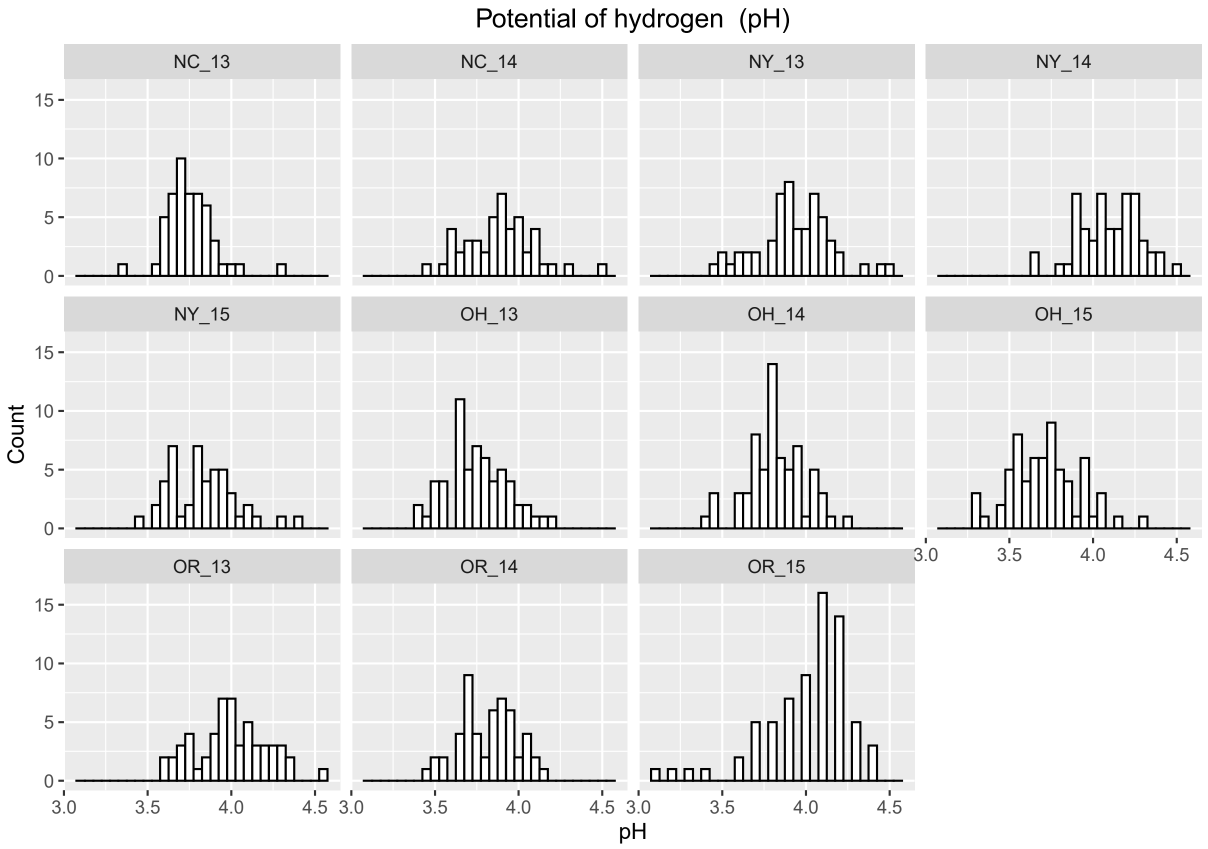

Figure S8. Distributions of pH in eleven location-by-year environments.

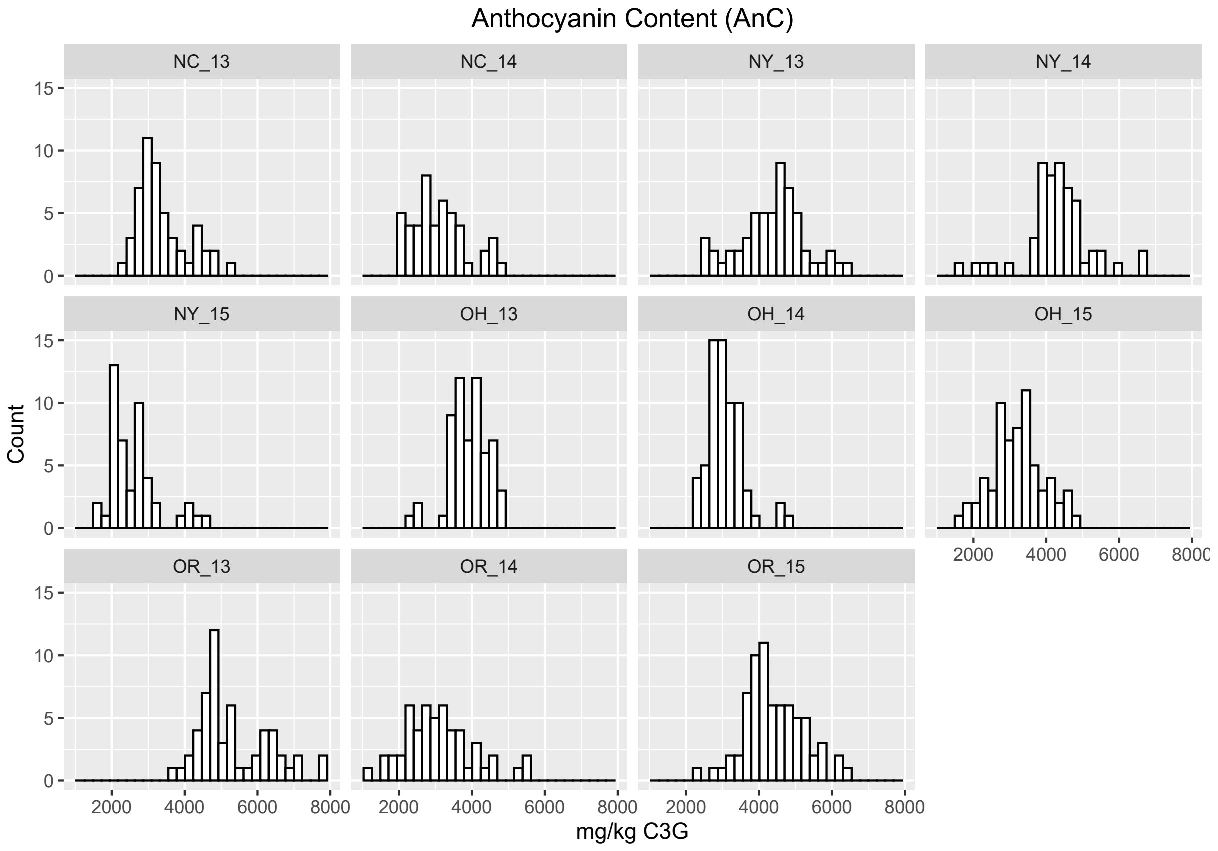

Figure S9. Distributions of anthocyanin content observed in eleven location-by-year environments. C3G=cyanadin 3-glucoside.

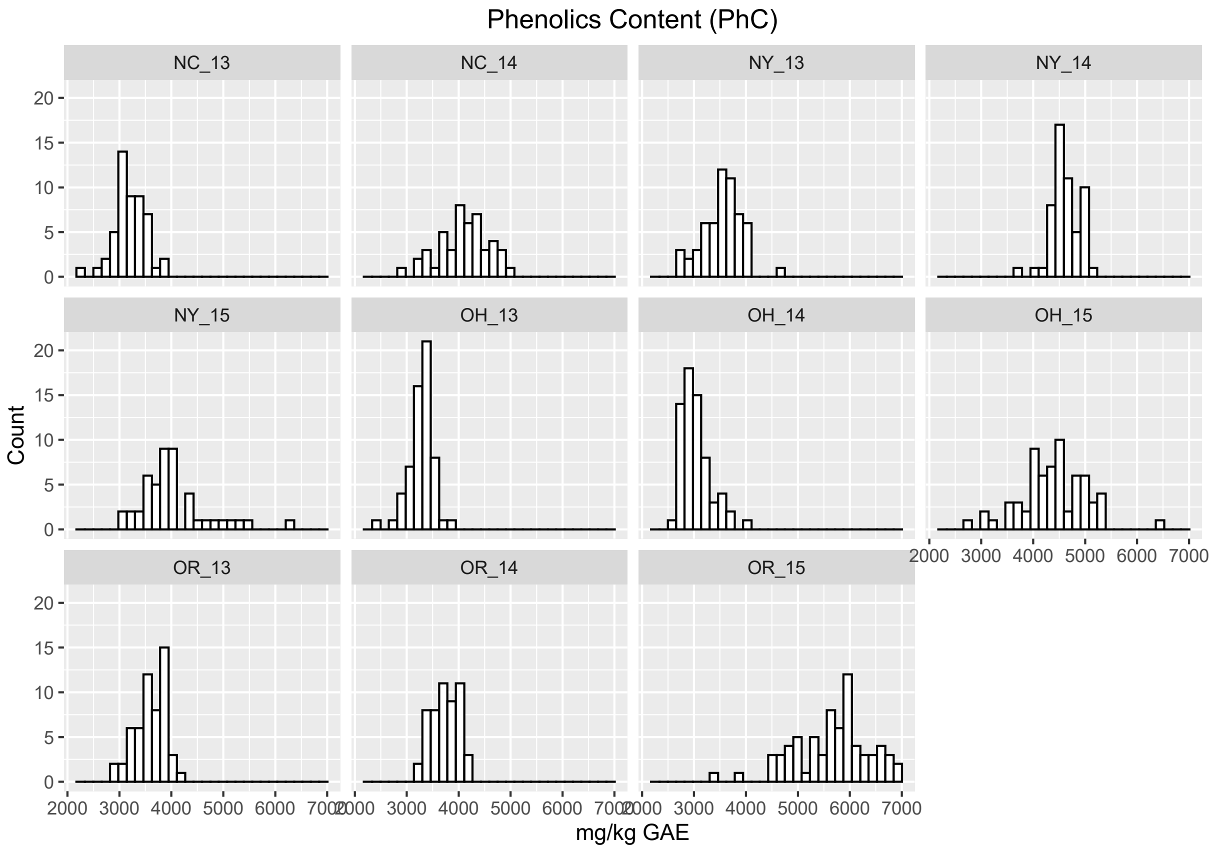

Figure S10. Distributions of phenolics content observed in eleven location-by-year environments. GAE=gallic acid equivalents.

Table S1. Number of ORUS 4305 individuals with phenotypic data for fruit size (FrM, SdM, DCt, DrM, SdF) and fruit chemistry (SSC, TAc, pH, AnC, PhC) within trial environments. In NC_13, fruit size data were available for 57 individuals and fruit chemistry data were available for 51 of 103 individuals used to construct linkage maps. Among all environments, fruit size data were available for 84 individuals, and fruit chemistry data were available for 80 individuals of 103 individuals used to construct linkage maps.

	**NC_13**	**NC_14**	**NC_15**	**NY_13**	**NY_14**	**NY_15**	**OH_13**	**OH_14**	**OH_15**	**OR_13**	**OR_14**	**OR_15**	**All**
**Size**	57	55	-	55	59	55	62	64	67	51	-	71	84
**Chemistry**	51	47	-	56	55	47	60	66	66	55	52	70	80
